# Comparative genomics reveals carbohydrate enzymatic fluctuations and herbivorous adaptations in arthropods

**DOI:** 10.1016/j.csbj.2024.10.027

**Published:** 2024-10-18

**Authors:** Dairon Ojeda-Martinez, Isabel Diaz, M. Estrella Santamaria, Félix Ortego

**Affiliations:** a Centro de Biotecnología y Genómica de Plantas, Universidad Politécnica de Madrid (UPM) – Instituto Nacional de Investigación y Tecnología Agraria y Alimentaria (INIA/CSIC) Campus de Montegancedo, Pozuelo de Alarcón, 28223 Madrid, Spain; b Departamento de Biotecnología-Biología Vegetal, Escuela Técnica Superior de Ingeniería Agronómica, Alimentaria y de Biosistemas, Universidad Politécnica de Madrid, Madrid, Spain,; c Centro de Investigaciones Biológicas Margarita Salas (CIB-CSIC), Ramiro de Maeztu 9, 28040 Madrid, Spain

**Keywords:** Comparative genomics, CAZome, Carbohydrate metabolism, Evolution, Arthropoda, Ancestral-state reconstruction, Herbivory

## Abstract

**Background:**

Arthropods represent the largest and most diverse phylum on Earth, playing a pivotal role in the biosphere. One key to their evolutionary success is their ability to feed on plant material. However, their endogenous enzymatic repertoire, which contributes to plant digestion, remains largely unexplored and poorly understood.

**Results:**

We analyzed 815 arthropod proteomes and identified a total of 268,171 carbohydrate-active modules. Our findings revealed a strong correlation between enzymatic content and feeding habits, with herbivorous species possessing significantly higher enzyme levels. We identified widespread carbohydrate-active families across the AA, CBM, GH, and GT classes, and observed a progressive increase in taxa-exclusive families in more recent arthropod lineages. Notably, we highlighted the impact of the transition from ametabolous to holometabolous development on carbohydrate metabolism, as well as the ecological adaptations of different species groups. By reconstructing the ancestral enzymatic profiles of arthropods, we identified significant fluctuations in 10 carbohydrate-active families over time.

**Conclusions:**

Our analysis advances the understanding of the evolutionary mechanisms utilized by the megadiverse phylum Arthropoda. We emphasize the critical role of herbivory as a selective force shaping enzymatic strategies, particularly those involved in carbohydrate metabolism. The distribution and exclusivity of carbohydrate-active families across different arthropod groups provide insights into their evolutionary trajectories and offer a clearer picture of the metabolic pathways that led their ancestors to their present forms.

## Graphical Abstract

**Figure:**
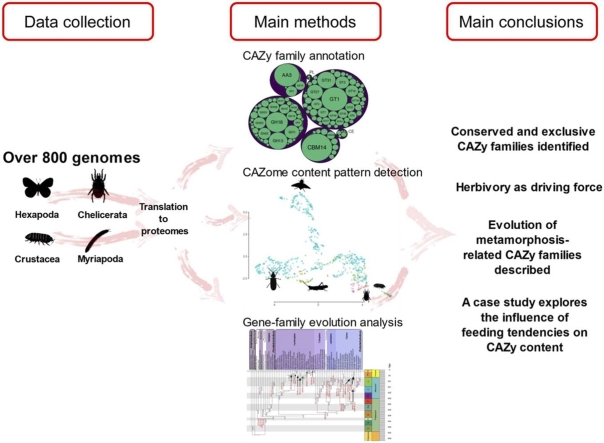

## 1 Introduction

CAZys (carbohydrate-active enzymes) are protein classes responsible for metabolizing all carbohydrates on Earth [1]. They play key roles in carbohydrate anabolism (via glycosyltransferases [GTs]), catabolism (via enzymes such as auxiliary activities [AAs], carbohydrate esterases [CEs], glycoside hydrolases [GHs], and polysaccharide lyases [PLs]), and carbohydrate binding (via carbohydrate-binding modules [CBMs]), and are found in all living organisms [2]. These enzymes are involved in critical physiological processes, including carbohydrate metabolism and digestion, detoxification of secondary metabolites, defence against pathogens, development, and other processes. Plant cell wall-degrading enzymes (PCWDEs), a subset of CAZys, belong to the AA, CE, GH, and PL classes. They degrade all major components of the plant cell wall via cellulases, hemicellulases, ligninases, and pectinases, which target cellulose, hemicellulose, lignin, and pectin, respectively [3]. Generally, oxidative enzymes involved in lignin degradation and lytic polysaccharide monooxygenases (LPMOs) responsible for cellulose decomposition are part of the AA class. Pectin acetylesterases and methylesterases fall under the CE class, while pectin and pectate lyases are members of the PL class. Hydrolases targeting hemicellulose, cellulose, or pectin are classified under GHs [4].

The ability of arthropods to digest and thrive on plant material has been a key factor in their success across various ecosystems. Most herbivorous arthropods, regardless of their degree of herbivory, produce endogenous CAZys that participate in the detoxification of plant secondary metabolites and in carbohydrate metabolism, including PCWDEs that break down plant cell walls [4]. This enzymatic capacity gives them an advantage, allowing them to exploit plant material in conjunction with other catalytic sources, such as fungi and bacteria, to thrive on otherwise impenetrable substrates [5]. The development of this ability arose through the long evolutionary interaction between arthropods and plants, spanning hundreds of millions of years. Although early arthropods are believed to have been detritivores [6], their adaptation to exploit a broader range of plant materials evolved rapidly [7]. The shift towards plant-based diets initiated complex interactions that set both plants and herbivores on an ongoing evolutionary battle for survival. In this context, the appearance of lignocellulose marked a significant turning point for both plants and their consumers [8]. The lignification of secondary cell walls made the degradation and utilization of plant material more difficult. Since lignin first appearence in plant biochemistry, it has remained a key component in plant biology, shaping plant-herbivore interactions. Over millions of years, plants developed numerous defence mechanisms and increased the structural complexity of lignocellulose [9]. The diversification of lignocellulose’s structure, polymer composition, linkage strength, and pore size contributed to its current variability and recalcitrance [10]. In parallel, arthropods evolved a range of strategies to cope with these changes, compensating for their inability to fully degrade plant material to monomers [11], [12], [13].

Throughout evolution, arthropods have developed numerous strategies—genetic, metabolic, behavioural, and mutualistic—that have enabled them to exploit plant-based resources. One such mechanism is horizontal gene transfer (HGT), through which arthropods have acquired the ability to degrade lignocellulose [14]. Increasing evidence suggests that arthropods have frequently obtained genes from bacteria or fungi via HGT [15]. The transfer of cellulolytic enzymes has been documented in groups such as coleopterans and phasmatodeans [4]. In both cases, these acquired enzymes have facilitated their adaptation to and exploitation of plant material as a food source [16], [17]. However, once introduced into a foreign genetic and metabolic environment, these enzymes are subject to various processes, such as duplications or mutations, which may lead to the loss or gain of functions [18], [19]. These events of neofunctionalization can also occur in endogenous lignocellulolytic enzymes, either narrowing or expanding their functionality, thus enabling arthropods to adapt to broader ranges of host plants [17], [20].

Another key strategy that allows arthropods to consume plant material involves mutualistic associations with microorganisms. This is perhaps one of the most studied pathways through which arthropods have achieved their remarkable success. In these symbiotic relationships, lignocellulose degradation is accomplished at the holobiont level [21]. Termites are a well-known example of this interaction, as many species harbour microorganisms in specific gut regions, enabling them to effectively digest lignocellulose [22], [23]. Other arthropod groups, such as myriapods and terrestrial crustaceans, also rely on lignocellulose detritus, thanks to their symbionts. This reliance varies, from equal contributions by both host and symbionts, as seen in isopods [24] to a high dependence on symbionts, as is the case with myriapods [25]. Arthropods have also developed behaviours that indirectly enable them to degrade and consume plant material. Leafcutter ants, for example, practice herbivory by cultivating fungi on freshly cut plant material [26]. These ants maintain large-scale farming systems, utilizing the metabolic processes of the fungi, and primarily feed on the fungi's swollen hyphal cells [27].

Although many carbohydrolytic enzymes have been identified in arthropods, their lignocellulolytic mechanisms remain poorly understood. Key information about the specific enzymes and enzyme complexes in their genomes, their associations with developmental, taxonomic, or ecological traits, and how these enzymes have evolved over time is still largely unexplored. In this study, we analyse 815 arthropod genomes, focusing on their CAZomes and PCWDE-like repertoires. Our goal is to understand the relationships among feeding habits, development, taxonomy, and enzymatic strategies. This knowledge will shed light on the ancestral enzymatic reservoirs and the ecological characteristics of the species that possessed them. We examine relevant taxonomic groups and enzymes in detail to gain insights into the co-evolution of plants and arthropods. These findings contribute to addressing contemporary challenges, such as the development of more efficient lignocellulolytic mechanisms for biofuel production, the design of targeted biopesticides, and the conservation of species of interest, among other applications.

## 2 Results

### 2.1 Genome-wide CAZy comparisons

#### 2.1.1 CAZome volume across Arthropods

**Fig. 1:**
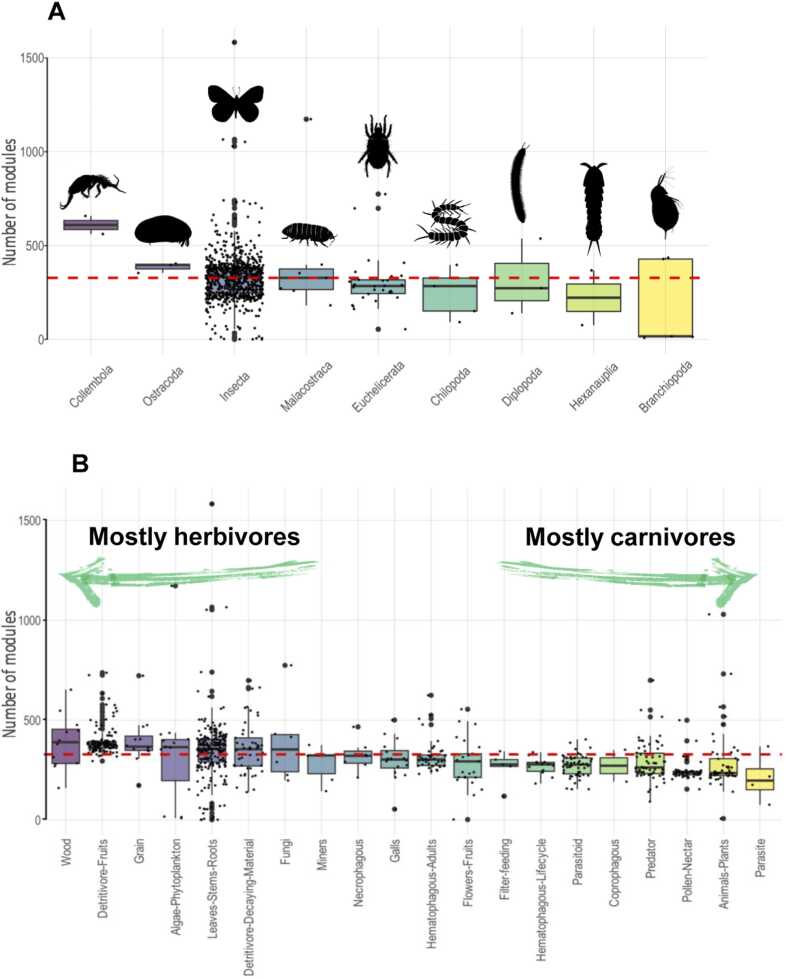
Box and whisker plots illustrating the distribution of CAZy module content. A, Plot showing the number of modules per species across different Arthropoda classes. B, Plot representing the number of modules per species across various feeding subclasses. In both plots, data is sorted in descending order based on the median values. The red dashed line indicates the overall median value of module content. Small dots represent individual species, while larger dots signify potential outliers. Only groups with more than two species are displayed. Illustrations were sourced from PhyloPic (http://phylopic.org/).

A total of 237,239 CAZy genes were identified across 815 arthropod species. However, since CAZys are modular in structure and can contain several modules with distinct individual functions, we will henceforth refer to them as modules. Using the dbCAN tool suite, we annotated 268,171 modules, distributed across the subphyla as follows: Hexapoda (250,893 modules across 759 species), Chelicerata (9612 modules across 31 species), Crustacea (5466 modules across 17 species), and Myriapoda (2200 modules across 8 species) (Supplemental file 1, Figs. S1A and S1B). To compare the number of CAZy modules per genome across subphylum and class, we tested multiple Generalized Linear Models (GLMs). Based on Model 1 from our study (Supplemental file 2), the number of CAZy modules per species varied significantly among subphyla (*χ*^2^_(3)_ = 11.67, p = 0.008) (Supplemental file 1, Fig. S1B). We observed a trend in which evolutionarily younger taxonomic classes (within Hexapoda and Crustacea) exhibited a higher number of CAZy modules per genome compared to their older counterparts, Chelicerata and Myriapoda (Fig. 1A).

The distribution of CAZy modules also varied according to feeding classes. Herbivorous species had the highest number of CAZy modules (124,322 across 379 species), followed by saprophagous (71,038 across 185 species), carnivorous (34,421 across 123 species), and omnivorous species (17,311 across 61 species) (Supplemental file 1, Fig. S2A). The feeding class “Other” (67 species), which includes a variety of feeding strategies, contained 21,079 CAZy modules. Model 1 also identified significant differences in CAZome content across feeding classes (*χ*^2^_(4)_ = 50.3, p < 0.001) (Supplemental file 1, Fig. S2B) and feeding subclasses (*χ*^2^_(13)_ = 44.0, p < 0.001) (Fig. 1B). A *post-hoc* pairwise analysis with Bonferroni correction followed the GLM analysis of feeding class and subclass data. This analysis revealed that arthropods with plant-based diets had significantly larger CAZomes compared to those with carnivorous or partially carnivorous diets (Fig. 1B, Supplemental File 2). A notable exception to this pattern was the group of pollen- and nectar-feeding arthropods, primarily represented by hymenopterans, whose median CAZy content was among the lowest, placing them alongside predominantly animal-based diet groups.

We also examined the conservation of mean CAZy content per genome across feeding subclasses. Standard deviation (SD) values of the mean CAZy content were calculated and bootstrapped 100,000 times to obtain Bias Corrected and Accelerated (BCa) Confidence Intervals (CIs) (Supplemental file 1, Fig. S3). The most conserved CAZomes (those with the lowest SD values) were found in hematophagous and parasitoid arthropods, as well as drosophilid fruit detritivores and pollen- and nectar-feeding hymenopterans. For pollen and nectar feeders, the CIs indicated variations as low as 27 to 84 enzymes per genome. In contrast, the least conserved CAZomes were observed in algae-, fungi-, and leaf-stem-root-feeding species, as well as in omnivores and detritivores.

#### 2.1.2 Distribution and abundance of CAZy classes

**Fig. 2:**
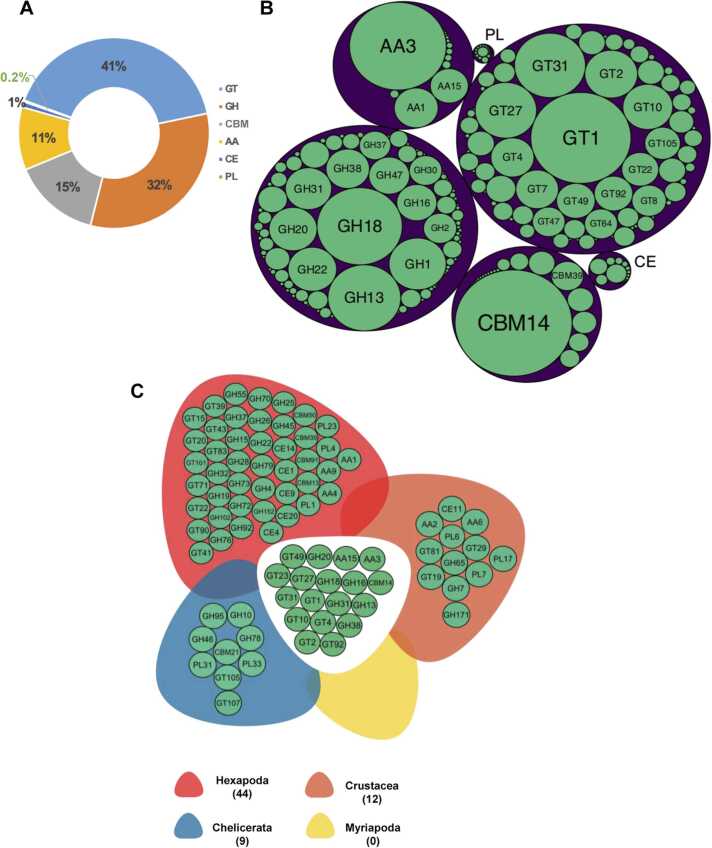
Plots representing the composition of CAZome classes and families across Arthropoda. A, Pie chart depicting the overall distribution of CAZy classes identified in the study. B, A circular packing plot showing the families identified across Arthropoda. Each purple circle represents a CAZy class, while green circles represent the corresponding families. The size of the circles indicates the number of components in each class or family. Only families with more than 2000 components are labelled in the circular plot. C, A diagram of exclusive and shared CAZy families having elevated duplication across Arthropoda subphyla. The leaves indicate exclusive CAZy families for each subphylum, while the centre shows families shared by all subphyla. Only CAZy families with at least 10 module copies per species are represented.

The analysis revealed the presence of all six CAZy classes (AAs, CBMs, CEs, GHs, GTs, and PLs), and a total of 306 CAZy families (Fig. 2A). Among these, the GT class was the most abundant across arthropod genomes, represented by 85 families and 110,347 modules, accounting for 41 % of the total CAZy modules. In contrast, the least abundant class was PL, with only 645 instances (0.2 %). The four most abundant families were CBM14, known for binding chitin (29,868 modules); the large GT1 family (21,912 modules), which plays a significant role in glycosylation processes; the widely represented oxidoreductase AA3 family (20,438 modules); and the chitinase family GH18 (15,851 modules) (Fig. 2B, Supplemental file 3, Table S1).

We evaluated the internal variation of CAZy families across the four subphyla of Arthropoda using heatmaps and hierarchical clustering. Detailed heatmaps for each CAZy class can be found in Supplemental file 1, Figs. S4–S5. To identify broader patterns of presence or absence, and to mitigate errors associated with potential mis annotations or genome quality, Fig. 2C presents only those families with at least 10 module copies per species. The four most prevalent families from the AA, CBM, GH, and GT classes were widely distributed across all arthropods. In addition to these, 14 other families formed a core set of CAZy families shared by all arthropods. Notably, Myriapoda was the only taxon without any exclusive CAZy families, while an increasing number of exclusive families were observed in younger subphyla.

Hierarchical clustering of CAZy module counts per species was performed to reveal broad patterns of enzyme availability within their genomes (Supplemental file 4). Generally, species clustered according to enzyme content in alignment with their taxonomic groups. With few exceptions, species from the orders Lepidoptera and Diptera clustered closely together, showing tighter groupings compared to species from other orders. Several coleopteran and hemipteran species also clustered near one another, while hymenopterans displayed more dispersed clusters, sometimes grouping with species from other orders.

#### 2.1.3 CAZy family compositions and their link to feeding, development, and taxonomy

**Fig. 3:**
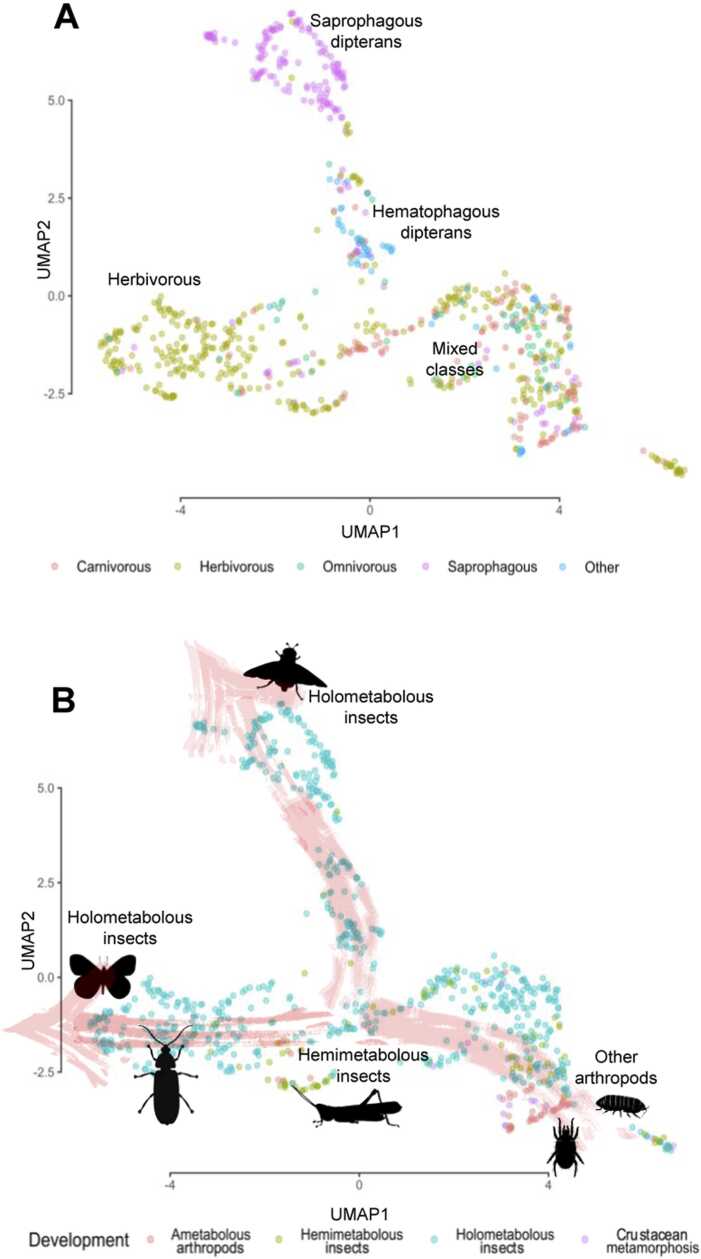
UMAP projection showing the distribution of CAZy families across the studied arthropod species. A, Colours represent different feeding preferences, highlighting the divergence in dipteran enzymatic strategies. B, Colours indicate developmental changes related to metamorphosis, with red arrows showing two main trends in enzymatic strategies among holometabolans. UMAP was constructed using the following parameters: Minimum distance = 0.1, and Number of neighbors = 17. Illustrations were sourced from PhyloPic (http://phylopic.org/).

To further investigate the underlying patterns of enzymatic diversity, we performed a Uniform Manifold Approximation and Projection (UMAP) analysis (Fig. 3). This analysis provided a spatial projection of all species based on the number of CAZy families present in their genomes. We examined the relationships between CAZy content and various traits, including feeding habits, taxonomy, and developmental stages. Notably, a distinct pattern emerged regarding feeding habits. The plot displayed a tight cluster of species with mixed feeding habits, from which distinct groups of herbivorous species diverged further to the left (Fig. 3A). The divergence in enzymatic strategies driven by feeding preferences became even more pronounced when focusing on specific cases within Diptera. For instance, clear differences were observed between hematophagous mosquitoes and saprophagous (herbivorous) drosophilids. Notably, drosophilids exhibited the exclusive presence of the modules GH43, CE5, PL26 and PL4, which were absent in mosquitoes (Supplemental file 3, Table S1).

Developmental and taxonomic patterns were also evident (Fig. 3B). A tight cluster of ametabolous arthropods, including chelicerates, myriapods, collembolans, and crustaceans, was followed by regions dominated by hemimetabolous insects. From this point, the enzymatic repertoires of holometabolous insects diverged, with lepidopterans and coleopterans clustering towards the left, while dipterans moved upwards. The overall pattern aligned with the evolutionary progression of these species, showing older arthropod groups clustered in the lower-right corner of the plot, with younger groups branching out toward the left. A more detailed UMAP plot, integrating both developmental and taxonomic criteria for each species, is available in Supplemental file 1, Fig. S6.

#### 2.1.4 Putative PCWDEs and lignocellulolytic AA families

**Fig. 4:**
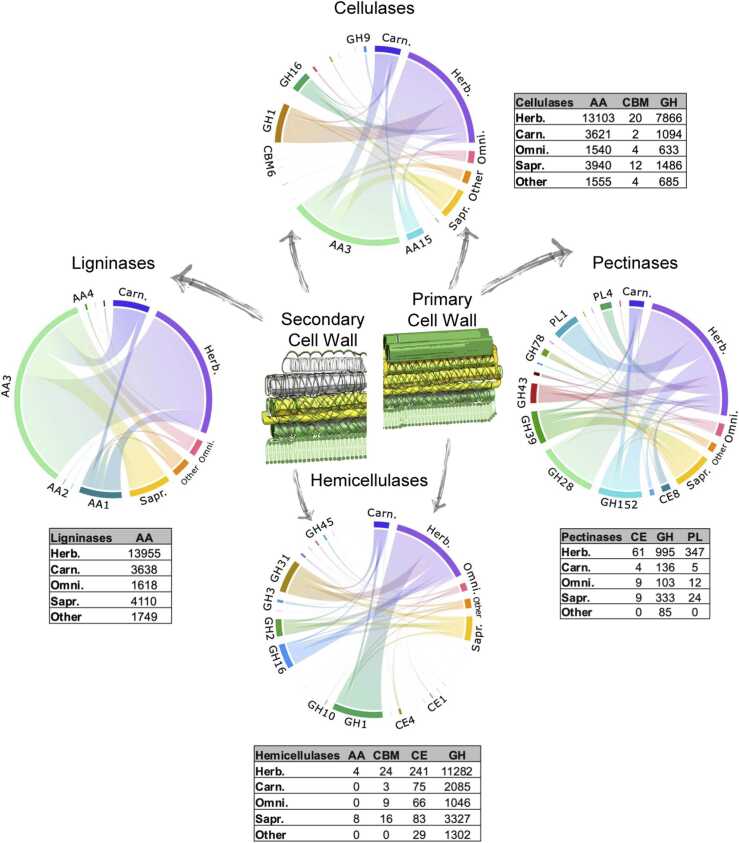
Distribution of PCWDE-like modules across dietary habits in arthropods. Chord plots illustrate the distribution of CAZy families associated with PCWDE-like modules across different dietary classes of arthropods. The tables provide a breakdown of the total count of each putative enzyme group per CAZy and dietary class. PCWDE-like modules include enzymes that act on plant cell wall polymers, as well as those that target monomers and oligomers. Enzymes with accessory functions crucial for plant cell wall degradation, such as those involved in producing reactants for lignocellulolytic pathways and detoxifying compounds, were also included in the relevant families and classes. Dietary class abbreviations: Herb., Herbivorous; Carn., Carnivorous; Omni., Omnivorous; Sapr., Saprophagous.

Building on our previous findings that linked herbivory to larger CAZomes and distinct CAZy repertoires, we aimed to determine whether these changes specifically involved PCWDEs. To do so, we identified and extracted from the CAZomes those modules potentially responsible for cell wall degradation. Of the 306 CAZy families identified, 91 were classified as putative cell wall-degrading enzymes (CWDE-like) derived from plants, fungi, or bacteria. From this subset, the contents of the 64 families classified as PCWDE-like were compared across feeding classes (Fig. 4). Herbivores exhibited significantly higher PCWDE counts compared to other feeding classes, as confirmed by a GLM followed by pairwise analysis (*χ*^2^_(4)_ = 109.8, p < 0.001) (Supplemental file 1, Fig. S7A; Supplemental file 2). The distribution of PCWDE-like families across feeding classes showed the prevalence of AA and GH members (Supplemental file 1, Fig. S7B).

**Fig. 5:**
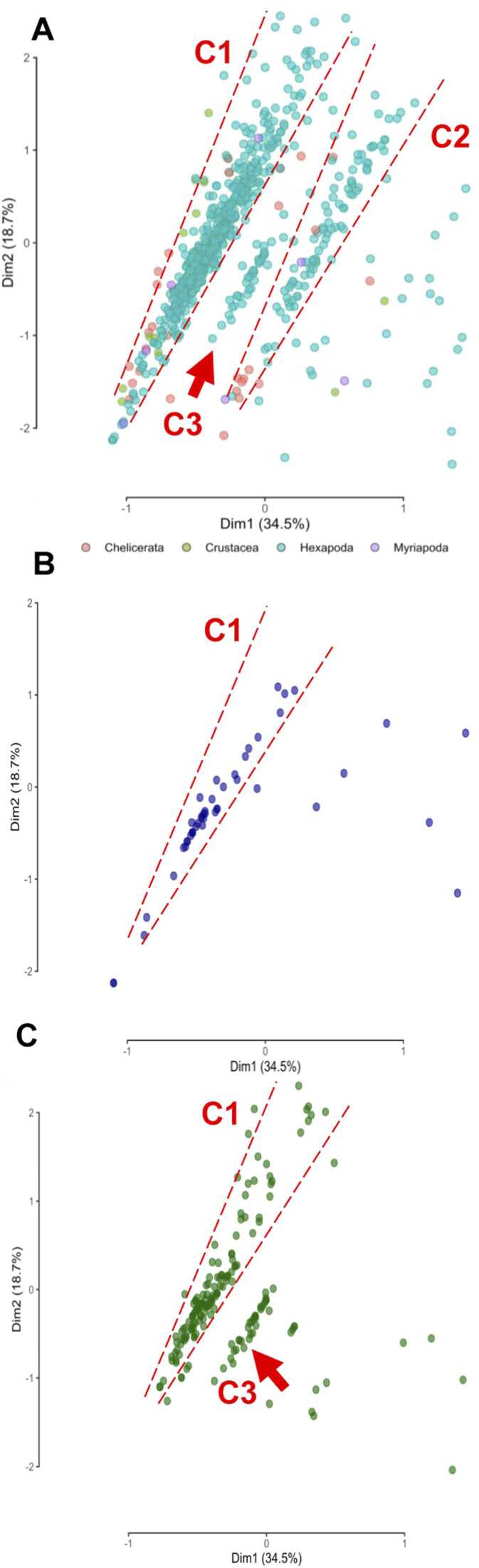
PCA detailing the distribution of selected AA CAZy families across arthropods. A, PCA projection of species, with red dashed lines highlighting two funnel-like clusters (C1-C2) and an intermediary cluster (C3). B, A close-up view of hemipterans, which form a distinct cluster (C1) unlike other arthropods. C, A focus on hymenopterans, which form an intermediary cluster (C3) represented by a red arrow, unique to this order.

Given the prominence of AA members, we further examined the distribution of this class across arthropod taxa. The AA class includes some of the most active enzymes involved in lignocellulose degradation, which function through oxidation-reduction reactions. It is also the only class that contains enzymes capable of breaking down lignin, a key polymer that influences plant-insect interactions. To explore the distribution of the AA class among arthropods, we used the number of identified AA CAZy families per species in a Principal Component Analysis (PCA). The Kaiser-Meyer-Olkin (KMO) test, determinant value, and Bartlett’s test results (Supplemental file 2) were employed to extract the most informative AA families for this analysis. The first two components, derived from AA families AA1 to AA4, AA6, AA7, AA10, and AA15, explained 53.2 % of the overall variability (Supplemental file 1, Fig. S8). To avoid the impact of isolated outliers, a close-up of the tightly clustered core species was conducted. Based on their AA content, species formed two funnel-shaped clusters (C1 and C2) in the plot (Fig. 5A). The tips of these funnels were densely packed with non-insect species, closely related to each other. From these regions, clusters of insect species radiated outward. Notably, hemipterans formed a single distinct cluster (C1) (Fig. 5B), while other hexapodan orders were distributed across both clusters. Hymenopterans, in addition to forming part of C1, created an intermediate cluster (C3) dominated by pollen- and nectar-feeding species (Fig. 5C). These patterns suggested an increase in the number and diversification of AA components in arthropod genomes, particularly among younger insect groups.

**Fig. 6:**
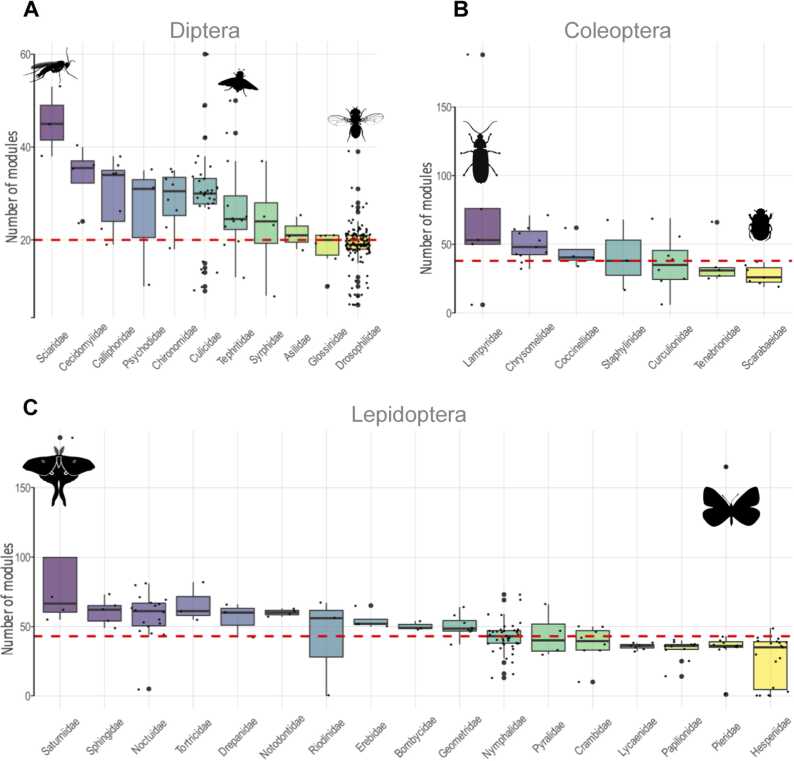
Box and whisker plots showing the distribution of AA class CAZy modules. A, Distribution of modules across families within Diptera. B, Distribution of modules across families within Coleoptera. C, Distribution of modules across families within Lepidoptera. Each plot is sorted in descending order based on the median value of ligninolytic and auxiliary CAZy modules (AA1-AA8). The red dashed line represents the overall median value of module frequency. Small dots indicate individual species, while larger dots denote potential outliers. Only families with more than two species are shown. Illustrations were sourced from PhyloPic (http://phylopic.org/).

We further investigated the significance of AA content among key herbivorous taxa. Box and whisker plots were generated to assess the number of putative ligninolytic AA families (AA1 and AA2) and accessory AA families (AA3 to AA8) across selected taxa. The orders Diptera, Coleoptera, and Lepidoptera were chosen due to the suggestion that herbivory contributed to their divergence from the enzymatic genome content of other arthropods. The analysis focused on the putative AA ligninolytic content within these groups. Among decaying wood-feeding dipterans, the Sciaridae family exhibited the highest median values, while drosophilids had the lowest (Fig. 6A). In Coleoptera, the Lampyridae family led with the highest median, whereas scarabs displayed notably low values (Fig. 6B). Within Lepidoptera, Saturniidae members showed the highest median, with *Actias luna* displaying exceptionally high values (Fig. 6C). In contrast, Hesperiidae and Pieridae were at the lower end, although the pierid *Delias pasithoe* exhibited unusually high AA content in its genome (Fig. 6C).

### 2.2 Gene-family size evolution of CAZy families in Arthropoda

Given the observed CAZy variations across taxa, as well as ecological and biological traits, we sought to uncover the evolutionary dynamics behind these enzymes. Our goals were to: determine the composition of the ancestral CAZomes of current species; identify significant events of CAZy family gains and losses, and pinpoint when they occurred; and relate these events to current biological traits. To achieve this, we implemented birth-death models on CAZy family count data using the CAFE software, which allowed us to model ancestral fluctuations in CAZy families.

**Fig. 7:**
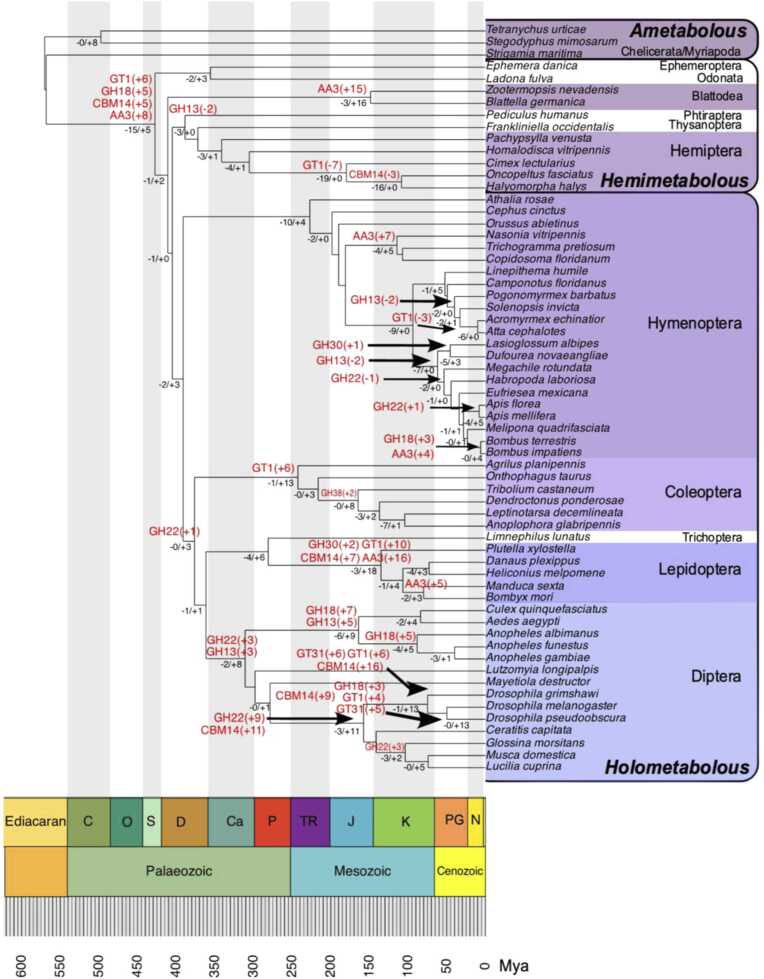
CAFE analysis on the evolution of the dynamics of CAZy gene families in Arthropoda. Ancestral fluctuations, including gene family contractions and expansions, were reconstructed for 62 species and are shown at the tree nodes in black text. Significant expansions or contractions of specific families at internal nodes are represented in red text. The phylogenetic tree’s topology and divergence dates are based on Thomas et al. (2020), and only species that overlapped between that study and ours were included in the analysis. Ancestral CAZy compositions were estimated based on the number of CAZy modules per species annotated in this study.

Due to the high variability in CAZy family content across Arthropoda, the models tested in CAFE struggled to converge. Multi-λ models also failed to achieve convergence. However, we obtained consistent results using the global-λ approach with the Base model on a Uniform root frequency distribution and the Gamma model on a Poisson or Uniform root frequency distribution. The best-fitting model for our data (-lnL = 6521.44) was based on a Gamma distribution with a Poisson root frequency distribution, utilizing two gamma rate categories (k = 2). This model enabled us to estimate CAZy content at internal nodes and detect gene families exhibiting rapid evolution. The overall λ value was 0.001192618, with a maximum possible λ of 0.00175953. Among the families analysed, 10 were identified as having significant evolutionary fluctuations: AA3, CBM14, GH13, GH18, GH22, GH30, GH38, GT1, GT27, and GT31. Significant expansions and contractions of these families across species, as well as fluctuations at internal nodes, are illustrated in Fig. 7. A comprehensive list of the ancestral CAZy compositions of the analysed species can be found in Supplemental file 3, Table S2, with node identities detailed in Fig. S9.

Our analysis revealed that the first major introduction of multiple enzymes occurred in the ancestor of hemimetabolous insects, marked by a significant increase in the number of enzymes from the families AA3, CBM14, GH18, and GT1. This coincides with the transition from ametabolous to hemimetabolous development and the associated metabolic shifts. In holometabolous insects, a gradual enrichment of GH22 enzymes characterized Diptera, with an increasing trend in brachyceran species. However, the nematoceran branch within Diptera diverged through an increase in GH18 enzymes. Particularly noteworthy is the substantial increase in enzymes from multiple families in the ancestor of Lepidoptera, contrasting with groups like Hymenoptera and Hemiptera, which tended to progressively reduce their CAZy repertoires. Notably, the CAZy families AA3 and CBM14 exhibited significant expansions across a broad range of taxa.

## 3 Discussion

### 3.1 CAZy content patterns point to essential metabolic and developmental processes

The composition of the identified CAZy classes provided insight into the broad makeup of arthropod CAZomes. The two dominant classes, GT and GH, are generally associated with fundamental carbohydrate metabolism [28]. Together, they accounted for 73% of all arthropod CAZys, a proportion similar to findings from a metagenomic study reporting over 80% for these two classes [29]. The similarity in these proportions between microorganisms and metazoans, despite their divergent ecosystems and biological differences, highlights the essential role of these classes in carbohydrate metabolism across diverse life forms. GT enzymes, in particular, mediate the transfer of sugar moieties [30], with GT1 being the most prevalent family in our data, involved in glycosylation of essential molecules like steroids, terpenes, and cofactors [31].

Alongside GT and GH, three other highly prevalent families were CBM14, AA3, and GH18. CBM14 chitin-binding modules are found across all domains of life [32], and are known to form part of the peritrophic matrix in the guts of species like termites [33]. The AA3 family, which comprises glucose-methanol-choline (GMC) oxidoreductases, is extensively present in fungi [34], and isopods [35], although its biological roles remain not fully understood. Lastly, GH18, previously reported as widespread in arthropods, includes chitin catalytic domains that perform vital functions such as immune defence, development, and tissue remodelling [36], [37].

The four most numerous CAZy families (AA3, CBM14, GH18, and GT1), along with 14 others, were widely present across all arthropod subphyla, indicating their critical role in basal metabolism. However, the number of these core enzymes is not static in arthropod genomes. Our gene-family size evolution analysis revealed significant fluctuations in these enzyme numbers over time. Notably, of the 10 fluctuating families identified by CAFE, all except GH22 and GH30 belonged to this core group of common CAZys.

A particularly significant finding was the increased presence of enzymes from AA3, CBM14, GH18, and GT1 coinciding with the evolutionary transition from ametaboly to hemimetaboly. This shift introduced a more distinct nymph stage, the development of wings, and flight, leading to changes in postembryonic development [38]. Nymphs, unlike adults, have wing pads instead of fully developed wings and possess a different cuticle [39], reflecting the metabolic adjustments required to accommodate these changes. The UMAP analysis further confirmed the metabolic divergence, placing hemimetabolous insects in an intermediary state between ametabolous and holometabolous species.

Most significant CAZy rate variations modelled over time occurred in holometabolous species. The transition to holometabolous development introduced a larval stage capable of exploiting different food sources, while morphologically distinct adults became specialized in dispersal and reproduction [38]. Our analysis showed that basal holometabolan groups, such as hymenopterans, retain CAZy configurations similar to those of hemimetabolous insects. In contrast, higher holometabolans, including Lepidoptera, Coleoptera, and Diptera, displayed clearly divergent carbohydrate metabolism strategies, as revealed by UMAP. The CAFE results pinpointed the specific CAZy families that fluctuated and the timing of these changes, which were particularly pronounced in Lepidoptera and Diptera. These findings reflect the significant impact metamorphosis had on genomic resources, driving divergent enzymatic approaches, either through increased quantity, enhanced functionality, or both. This genomic plasticity contributed to the evolutionary success of Arthropoda, enabling them to adapt and thrive.

### 3.2 Herbivory as driving force for CAZy acquisition

The progressive increase in taxa-specific CAZy families observed in our data aligns with the evolutionary history of arthropods and their feeding habits. The detritivorous ancestor of arthropods, emerging early in evolutionary history, already possessed a set of enzymes for degrading algae. These enzymes were inherited from its bilaterian predecessor and are also found in older groups such as Annelida, Cnidaria, Nematoda, and Mollusca [7]. The native enzyme repertoire evolved through gene duplications, mutations, and contractions. Additionally, enzymes involved in metabolic pathways underwent neofunctionalization events, enabling arthropods to diversify their feeding strategies. This evolutionary flexibility was partly driven by the inherent plasticity of these catabolic enzymes, which can act on a broad spectrum of substrates [40]. These enzyme acquisitions not only facilitated the depolymerization of plant cell wall components but also enabled the detoxification of secondary metabolites through various mechanisms [7]. Mutualistic relationships between arthropods, fungi, and bacteria further enhanced their ability to degrade lignocellulose at the holobiont level. However, this interaction also caused fluctuations in the host's endogenous PCWDE repertoire [21], [35]. Dependence on exogenous enzymatic sources led to both genomic relaxation and enrichment processes, often driven by HGT events. HGT is notably more frequent in arthropods compared to other animal taxa and has played a crucial role in the evolution of herbivory in arthropods [14], [18].

Herbivorous and detritivorous species exhibited higher CAZy content compared to other feeding groups, with herbivores showing particularly elevated levels of PCWDE-like modules. Previous studies have linked higher CAZy and PCWDE content to an enhanced capacity for lignocellulosic degradation, as seen in fungal organisms [41]. The allocation of genetic material toward specific functions serves as direct evidence of their significance, given the limitations associated with genome resources [42], [43]. Our data suggest that herbivory necessitates a substantial investment of genetic resources in arthropods, comparable to factors such as body size and developmental processes [44]. In contrast, animal-feeding species displayed significantly lower CAZy and PCWDE-like contents. Unlike herbivores, animal-feeding species tend to consume more proteins and fats but smaller amounts of carbohydrates. Consequently, they possess less complex and less diverse enzymatic repertoires for carbohydrate degradation. Parasitic species, which closely resemble carnivorous CAZome counts, often become increasingly dependent on their hosts' metabolism, focusing on scavenging nutrients rather than processing them [45]. In line with this, our bootstrapped confidence interval (CI) results indicated that CAZome counts were highly conserved among parasitic species. Conversely, arthropods that feed on plant material not only exhibited numerous and varied enzyme ensembles but also demonstrated greater heterogeneity in their enzymatic counts within the same feeding category. This pattern suggests that herbivores experience heightened selective pressure on carbohydrate metabolism, resulting in increased variability in their enzymatic resources.

Our exploration of the AA class, which includes ligninolytic families, allowed us to investigate the relationship between herbivory and CAZy content more deeply. Lignin evolved in plants approximately 450–400 million years ago, during the late Ordovician to early Devonian periods [8]. At that time, the earliest hexapod taxa were differentiating, while most, if not all, ancestors of chelicerates, myriapods, and crustaceans had already established themselves. Therefore, it is expected that the ligninolytic enzymatic contents of these established groups would remain relatively rudimentary. Our PCA results supported this idea, revealing that non-hexapod species had similar AA contents. Furthermore, the ligninolytic strategies of hexapods were distinct from those of their non-hexapod counterparts, reflecting diversification and an increase in the number of ligninolytic enzymes within these species. Notably, current understanding indicates that the complete decomposition of lignin to CO_2_ and H_2_O is restricted to white rot fungi [8]. However, members of the AA class can act on a broad spectrum of phenolic compounds beyond lignin, allowing them to detoxify secondary metabolites from plants and thus facilitating access to more complex plant material [46], [47]. The constitutive expression of these enzymes in the Malpighian tubules and midgut of arthropods suggests their roles in both detoxification and digestion processes [48]. Additionally, the enrichment of AA members aligns with the increased developmental complexities observed in hemimetaboly and holometaboly. The plasticity of these enzymes makes them essential candidates for participating in the more complex cuticle processes related to development in these species [49]. Exceptions among insects are hemipterans, which exhibit simpler ligninolytic strategies. Their feeding style, involving piercing and sucking plant material, allows them to avoid consuming lignin-rich tissues. Moreover, hemipterans have lost several digestive enzyme groups throughout their evolution [6], a finding consistent with our CAFE results, which indicated progressive contractions in enzyme family members for these species.

The variability in enzymatic strategies among hexapods was closely linked to their feeding habits. Coleoptera, with its diverse range of feeding styles, exhibited the most diverse enzymatic strategies. In contrast, Lepidoptera species showed much more homogeneity in their enzymatic strategies, which aligns with their predominantly herbivorous diet. At the other extreme was Diptera, where two distinct enzymatic strategies emerged, corresponding to two feeding tendencies: fruit-decaying saprophagous drosophilids and animal-based feeders like hematophagous mosquitoes. Our data highlighted the nature of this divergence, which involved PCWDE-like modules and other CAZys. It revealed the presence of PCWDE-like modules such as GH43, CE5, PL26 and PL4 in drosophilids but not in mosquitoes. Regarding CAZys not classified as PCWDEs, the phylogenetic analysis showed a progressive increase in GH22 modules and several other enzymes in drosophilids, while mosquito proteomes displayed significant increases in GH13 and GH18 members. The enzymatic variation within Diptera, especially between these two groups, underscores the idea that feeding on plant material drives divergence in enzymatic resources. In both this case and that of Lepidoptera, the near-exclusive focus on herbivory shaped their enzymatic strategies. As these examples illustrate, herbivory exerts evolutionary pressure on genetic resources, leading to distinct enzymatic strategies both among and within closely related taxa.

An important consideration in this study is that PCWDE-like modules were annotated based on CAZy patterns in well-studied organisms such as bacteria and fungi. Since CAZys are classified based on amino acid sequence similarities, members of a family typically share similar protein folding and catalytic mechanisms [34]. However, the biological function of CAZy families can vary depending on the tissue or developmental stage in which they are expressed. The annotation of PCWDE-like modules in non-herbivorous species also suggests their involvement in other biological processes. This functional divergence may result from their inherent multifunctionality or through processes such as neofunctionalization. In fungi, members of the same CAZy family can participate in multiple functions or metabolic pathways. This pattern has also been observed in arthropods, as in the case of AA3 families, which are involved in cuticle development, detoxification of plant secondary metabolites, and the production of reactants for lignocellulolytic reactions [34], [50], [51]. Therefore, in our annotation of these enzymes, we considered that the biological roles of CAZys may differ between fungi and arthropods.

### 3.3 Hymenopterans as a case-study of feeding habits influencing CAZy content

Throughout our analysis, several results highlighted distinct characteristics related to the order Hymenoptera. The CAZomes of hymenopterans were unusually smaller compared to their herbivorous counterparts, their enzymatic strategies across feeding subgroups were remarkably conserved, they frequently exhibited enzyme family contractions, and they displayed exclusive AA ensembles. These characteristics may be explained by the evolutionary history of this group. Although early hymenopterans were phytophagous, their shift towards a predominantly parasitoid lifestyle is estimated to have occurred in the late Triassic and has remained the dominant mode of life [52]. As parasitoids must adapt to the metabolism of their hosts, leading to increased specialization, their evolutionary potential is restricted, which in turn hampers diversification [53]. This transition likely resulted in the simplification of both the digestive system and general metabolism in most hymenopterans [6], potentially explaining the reduced carbohydrate metabolism observed in our data.

Interestingly, members of the AA10 family, which are typically reported in fungi, prokaryotes, and some viruses, were detected in 33 species from the family Apidae. However, since this module is known to be produced by the pathogenic bacteria *Paneibacillus larvae* and entomopoxviruses (EVs), where it participates in the degradation of the chitin-rich peritrophic matrix (PM) of their hosts [54], we cannot definitively conclude whether these enzymes were acquired via HGT or whether the genome sequences analysed were contaminated with pathogen-derived sequences.

Following the simplified parasitoid model, a secondary shift toward phytophagy occurred in bees during the late Cretaceous [52], transitioning from parasitoidism to pollen and nectar feeding. The preference of bees for these simpler, less nutrient-rich sources may be a consequence of the metabolic simplification caused by parasitoidism, which created a genetic bottleneck. This process constrained their genetic resources, limiting them to simpler food sources. Compared to other plant materials, pollen and nectar are much less complex and contain lower levels of toxic secondary metabolites [55]. However, this shift toward an easier-to-digest food source also led to a "relaxation" in the capacity of these species to cope with toxic substances, resulting in a diminished detoxification system. Bees are known to have significantly lower levels of detoxification enzymes compared to other insects [56], [57].

Our analysis of the CAZomes of two strictly phytophagous families since their origin—Cephidae (one species, 267 modules) and Tenthredinidae (two species, 409 and 431 modules)—revealed that they had higher CAZome counts than the mean for Hymenoptera (259 modules). This finding supports the hypothesis that the parasitoid specialization within Hymenoptera led to a reduced and simplified carbohydrate metabolism. However, due to the limited sample size, further research on these and other extant phytophagous families is necessary to confirm this hypothesis, once more genomes become available. Although this reasoning may explain the evolutionary history of these species, the question remains as to why they reverted to herbivory and what evolutionary forces drove this change, given their limited resources to cope with such a shift.

## 4 Conclusions

The diversity of CAZome strategies in Arthropoda can be considered the result of multifaceted evolutionary processes. The gradual transformation of this vast group of organisms was driven by a combination of complex and powerful forces, shaping them into the forms we see today. One of the most significant factors in this transformation was the interaction between arthropods and plants, which exerted considerable pressure on their genetic resources, prompting adaptation and change. Equally important were interactions with microbiota, whether as partners or antagonists, as well as the influence of viruses and inter- and intraspecific competition. Our results highlight the impact of these pressures on the carbohydrate metabolism of arthropods, revealing a shared enzymatic heritage across taxa, along with the distinct evolutionary pathways each group followed to reach their current enzymatic profiles. Notably, our findings emphasize the pivotal role of herbivory as a major evolutionary force that shaped enzymatic strategies within Arthropoda. These dramatic evolutionary shifts underscore the remarkable plasticity of arthropod genetic resources—a characteristic that has been key to their success, but also in some cases, to their decline.

Many questions remain regarding the relative contributions of these various factors to the evolutionary transformations observed in arthropods. Ongoing evolutionary studies of this and other groups will continue to enhance our understanding of how life has evolved to its present state, and may even offer clues as to where it is headed.

## 5 Methods

### 5.1 Proteome retrieval and CAZy identification

CAZy identification was performed on proteomes obtained from multiple sources. Arthropod proteomes were downloaded according to availability, from the NCBI genome database [58], the InsectBase database [59] and the Orcae database [60]. Proteomes available on InsectBase and Orcae databases were downloaded and processed on “as is” basis. Proteomes from the NCBI database were subject to the following quality filters: only reference-annotated genomes were included, while atypical or contaminated genomes were excluded. For organisms without available proteomes, gene models were trained and predicted using Funannotate (v1.8.1) [61] based on their genomes, which were downloaded from the NCBI database. Detailed information on the species, their database of origin and accession number can be consulted on Supplemental file 3, Table S3.

Proteomes in FASTA format were processed using dbCAN3 [1], for CAZy identification, based on the Carbohydrate Active Enzyme (CAZy) database [62]. CAZys were classified into the following classes: Auxiliary Activities (AAs), Carbohydrate-Binding Modules (CBMs), Carbohydrate Esterases (CEs), Glycoside Hydrolases (GHs), Glycosyl Transferases (GTs) and Polysaccharide Lyases (PLs). CAZy annotation was automated using three tools with default E-value cut-offs: HMMER [63], DIAMOND [64] and dbCAN_sub [65]. CAZys identified by at least two of these three tools were selected for further analysis, as recommended by the developers.

### 5.2 Gene model prediction

Genomes downloaded from the NCBI database were processed to predict genes based on protein evidence, existing gene databases and ab initio predictors. Raw genomes were analysed using the software FastQC (http://www.bioinformatics.babraham.ac.uk/projects/fastqc/) and Trimmomatic (v. 0.38) [66] to assess quality and to clean adapters. Repetitive regions on the genomes were soft-masked using RepeatMasker (v. 4.1.5) [67]. The pre-processed genomes were then processed through the Funannotate pipeline (v1.8.1) [61] on the Galaxy platform [68] to predict gene structure. Prediction was done by this pipeline by the convergence of three different predictors: Augustus [69], glimmerHMM [70] and SNAP [71]. Protein evidence used to predict genes was based on UniProt/SwissProt. Training of ab initio predictors was performed by BUSCO (Benchmarking Universal Single-Copy Orthologs), aligning the models to the Arthropoda database and training Augustus using the fly database. Maximum (10,000) and minimum (30) intron lengths were modified to adapt to the Arthropoda genomes [72]. Repetitive regions were handled using EVM. Predicted protein-coding genes were searched with BLASTP against the UniProt database using DIAMOND, with a minimum identity threshold of 80 %. Genes with no similarity to known proteins or without database support were excluded from further analysis.

### 5.3 Taxonomic, feeding, development and PCWDE classifications

Taxonomic classification of the selected species (Supplemental File 3, Table S3) was performed using the taxize (v. 0.9.100) [73] package in R. This package classified species based on the taxonomic information from the Integrated Taxonomic Information System (ITIS) [74] database.

Feeding habits were categorized according to literature and expert opinion into five main classes, each with subclasses based on the species' primary food sources. The classes and subclasses were as follows: class Carnivorous, with subclasses Parasite, Parasitoid, and Predator; class Herbivorous, with subclasses Algae-Phytoplankton, Flowers-Fruits, Galls, Grain, Leaves-Stems-Roots, Miners, Phloem-Xylem, Pollen-Nectar, and Wood; class Omnivorous, with subclasses Animals-Plants and Filter-Feeding; class Saprophagous, with subclasses Detritivore-Decaying Material, Detritivore-Fruits, and Necrophagous; and class Other, with subclasses Animal-Secretions, Coprophagous, Fungi, Hematophagous-Adults, Hematophagous-Lifecycle, and Wax. Each subclass was named according to either their food source or trophic category (Supplemental File 3, Table S3). Saprophagous species were those that fed on decomposing organic material, while the class Other included species that consumed less common food sources. The subclass Animal-Secretions referred to species that feed on eye secretions, and the subclasses Hematophagous-Adults and Hematophagous-Lifecycle represented blood-feeding organisms, either exclusively in their adult stage (e.g., mosquitoes) or throughout their entire life cycle (e.g., Hemipterans), respectively.

Developmental classification regarding metamorphosis was based on taxonomic criteria [38]. All species of Hexapoda were classified according to their taxa-associated metamorphosis as either Ametabolous, Hemimetabolous, or Holometabolous. Chelicerate and Myriapod species were classified as Ametabolous. Crustaceans, which exhibit diverse developmental strategies—including some species without larval stages and others with significant morphological changes[75]—were classified under Crustacean Metamorphosis (Supplemental File 3, Table S3).

To identify which CAZy families were deemed as PCWDEs, previous studies that delved on the identification and classification of CAZys were consulted. A comprehensive list of CAZys considered as PCWDEs was obtained from [28], [76]. Further information on the role of each AA family member was obtained from [62]. The full list of the 91 enzymes deemed as putative PCWDEs, FCWDEs and/or BCWDEs from our CAZy annotation and their potential activities can be consulted on Supplemental File 3, Table S4.

### 5.4 Gene-family evolution analysis

Computational Analysis of gene Family Evolution (CAFE) was performed by using the software CAFE (v. 5) [77]. This software implements a birth-death model to estimate gene family evolution, i.e.: expansions and contractions of gene families. The estimations of family sizes and statistical analysis of variations provide a basis for analysing the significance of fluctuations in family size among taxa. To determine this information, an ultrametric tree with branch lengths expressed in time units and the number of CAZy per species were used. The topology and divergence dates of the phylogenetic tree were based on [78], only species coinciding between that study and ours were accounted for. CAZys not present in the selected species or showing low variability were filtered out by CAFE. This step left a total of 69 enzymes for the gene-family evolution analysis. An error model was calculated, to account for assembly and annotation errors across experiments and render better estimations of gene gain/loss.

A total of 21 models having various degrees of complexity were designed and tested to evaluate which of them best described our data (Supplemental file 2). Gene family evolution was estimated by employing either Uniform or Poisson distributions to describe the root frequency distribution, interacting with either the Base CAFE model or Gamma model to estimate gene family distribution. When Gamma distribution was used, an array of 1–10 gamma rate categories was tested. For all the models the Maximum Likelihood (ML) value of the parameter describing the probability of gene gain or loss (λ) was calculated, as well as the maximum possible λ for the whole tree topology. The model that achieved convergence and had the optimal ML value was used to test the hypothesis of the occurrence of different rates of gene family evolution depending on species taxa. To achieve this purpose, the results of the simple λ tree were compared to multiple λ trees: independent λ ´ s per subphylum, independent λ ´ s per order and independent λ ´ s per development type. The process of model analysis was repeated 400 times to ensure result convergence. Significance of gene family dynamics was determined as p < 0.05. From this information, CAFE was able to reconstruct ancestral gene family composition at internal nodes.

### 5.5 Statistical analyses

Dimensionality reduction was performed on number of CAZy data and AA subfamily count data. UMAP was used on CAZome count data due to its capability to express high dimensionality data on lower dimensions [79]. The analysis was done on the number of all the identified CAZy families and subfamilies per species. Calibration and identification of the optimum values of the two UMAP parameters “number of neighbours” and “minimum distance” was achieved by calculating a grid of their potential values. The grid covered five values from 0.1 to 0.9 in the case of “minimum distance” and five values from 3 to 22 in the case of “number of neighbours”. The range of selected values for the parameter “minimum distance” intended to sample its full range which is 0–0.99. The range of values selected for the parameter “number of neighbours” was based on the minimum and maximum of qualitative classifications of our variables (taxonomy-feeding subclasses). The combination of the parameter values that best resolved the underlying pattern was then selected to plot the results. CAZome count data was also used to produce heatplots and was processed by a hierarchical clustering method. Hierarchical clustering performed on count data was based on the Manhattan clustering distance, which accounts for count data that contains outliers [80]. Clustering distance was calculated using the Ward's hierarchical agglomerative clustering method [81]. Clusters formed were analysed under taxonomic criteria.

Principal Components Analysis (PCA) was done on the AA subfamily count data. Previous to PCA, various tests were done in order to measure the adequacy of each variable. A filtering process was done to select the most informative families based on Kaiser-Meyer-Olkin factor adequacy values. Families that had their Measure of Sampling Adequacy (MSA) below 0.5 were progressively left out of the final analysis, until the overall MSA reached an optimum value of 0.69. The determinant value and Bartlett’s test results were used as well to confirm the test adequacy. Those variables whose test values were not in accordance with the selected thresholds [82], were excluded from the analysis. The count data of the AA families selected was then plotted in a bidimensional area to determine patterns.

CAZome and PCWDE divergencies across taxa and feeding preferences were evaluated by applying Generalised Linear Models (GLM). For CAZome comparisons, the models were constructed having as dependent variable the number of modules and as factors: feeding preferences and the taxa criteria subphylum and class. Nine models were evaluated in increasing complexity of interactions among factors, excluding factor levels that had less than two cases. Criteria for model selection was based on AIC values, overdispersion test results and residuals behaviour. For each model, Poisson and negative binomial distributions were tested using a logarithmic link function. For PCWDE content comparisons, a GLM model based on a negative binomial distribution of PCWDE content across diet classes was used. The GLM results were further analysed using a pairwise comparison having Bonferroni correction to locate differences among groups. Models tested and their results can be consulted on Supplemental file 2.

CAZome conservation was evaluated based on bootstrapped SD of the mean CAZy content per genome. The SD values of the mean CAZy content were calculated and bootstrapped 100,000 times to obtain Bias Corrected and Accelerated (BCa) Confidence Intervals (CIs). Bootstrapped CI values were visualized using violin plots and fluctuations of the variability of genome overall count determined based on its values and plot behaviour.

For all tests, results having p values ≤ 0.05 were considered as significant. All statistical analyses were performed on the free software R (v. 4.3.1) [83].

## Funding

The authors gratefully acknowledge the Grants PID2020–112756RA-I00 and CNS2022–135194 funded by MCIN/AEI/10.13039/501100011033 and by 10.13039/501100000780European Union NextGenerationEU/PRTR. The “Margarita Salas” grant funded D.O.M. according to the RD 289/2021 and the order UNI/551/2021, funded by the program European Union NextGenerationEU/PRTR.

## CRediT authorship contribution statement

**Isabel Diaz:** Writing – review & editing, Funding acquisition, Conceptualization. **M. Estrella Santamaria:** Writing – review & editing, Funding acquisition, Conceptualization. **Dairon Antonio Ojeda Martinez:** Writing – review & editing, Writing – original draft, Methodology, Funding acquisition, Formal analysis, Data curation, Conceptualization. **Félix Ortego:** Writing – review & editing, Funding acquisition, Conceptualization.

## Declaration of Competing Interest

By this mean all authors declare no conflict of interest. This manuscript has not been submitted for publication elsewhere. All authors have read and approved the final version of the manuscript.

## Data Availability

All datasets generated and/or analysed during the current study are publicly available. All DNA, genome assemblies, and proteome assemblies can be found at the NCBI, the InsectBase database and the Orcae database. Information on accessions for all data including SRA accessions are listed in Supplemental file 3: Table S3. Any additional dataset used and/or analysed during the current study are available from the corresponding author on reasonable request.

## References

[1] Zheng J., Ge Q., Yan Y., Zhang X., Huang L., Yin Y. (2023). dbCAN3: automated carbohydrate-active enzyme and substrate annotation. Nucleic Acids Res.

[2] Huang L., Zhang H., Wu P., Entwistle S., Li X., Yohe T. (2018). dbCAN-seq: a database of carbohydrate-active enzyme (CAZyme) sequence and annotation. Nucleic Acids Res.

[3] Albersheim P., Darvill A., Roberts K., Sederoff R., Staehelin A. (2010).

[4] Tokuda G. (2019). Plant cell wall degradation in insects: Recent progress on endogenous enzymes revealed by multi-omics technologies. Adv Insect Phys.

[5] Bredon M., Dittmer J., Noël C., Moumen B., Bouchon D. (2018). Lignocellulose degradation at the holobiont level: teamwork in a keystone soil invertebrate. Microbiome.

[6] Terra W.R., Ferreira C. (2020). Evolutionary trends of digestion and absorption in the major insect orders. Arthropod Struct Dev.

[7] Calderón-Cortés N., Quesada M., Watanabe H., Cano-Camacho H., Oyama K. (2012). Endogenous plant cell wall digestion: A key mechanism in insect evolution. Annu Rev Ecol Evol Syst.

[8] Janusz G., Pawlik A., Sulej J., Świderska-Burek U., Jarosz-Wilkołazka A., Paszczyński A. (2017). Lignin degradation: microorganisms, enzymes involved, genomes analysis and evolution. FEMS Microbiol Rev.

[9] Ros Barceló A., Gómez Ros L.V., Gabaldón C., López-Serrano M., Pomar F., Carrión J.S. (2004). Basic peroxidases: The gateway for lignin evolution?. Phytochem Rev.

[10] MacDonald J., Doering M., Canam T., Gong Y., Guttman D.S., Campbell M.M. (2011). Transcriptomic responses of the softwood-degrading white-rot fungus *Phanerochaete carnosa* during growth on coniferous and deciduous wood. Appl Environ Microbiol.

[11] Kaoutari A.El, Armougom F., Gordon J.I., Raoult D., Henrissat B. (2013). The abundance and variety of carbohydrate-active enzymes in the human gut microbiota. Nat Rev Microbiol.

[12] Cragg S.M., Beckham G.T., Bruce N.C., Bugg T.D.H., Distel D.L., Dupree P. (2015). Lignocellulose degradation mechanisms across the Tree of Life. Curr Opin Chem Biol.

[13] Watanabe H., Tokuda G. (2010). Cellulolytic systems in insects. Annu Rev Entomol.

[14] Wybouw N., Pauchet Y., Heckel D.G., Leeuwen T., Van (2016). Horizontal gene transfer contributes to the evolution of arthropod herbivory. Genome Biol Evol.

[15] Husnik F., McCutcheon J.P. (2018). Functional horizontal gene transfer from bacteria to eukaryotes. Nat Rev Microbiol.

[16] Vega F.E., Brown S.M., Chen H., Shen E., Nair M.B., Ceja-Navarro J.A. (2015). Draft genome of the most devastating insect pest of coffee worldwide: The coffee berry borer, *Hypothenemus hampei*. Sci Rep.

[17] Shelomi M., Danchin E.G.J., Heckel D., Wipfler B., Bradler S., Zhou X. (2016). Horizontal gene transfer of pectinases from bacteria preceded the diversification of stick and leaf insects. Sci Rep.

[18] Kirsch R., Gramzow L., Theißen G., Siegfried B.D., ffrench-Constant R.H., Heckel D.G. (2014). Horizontal gene transfer and functional diversification of plant cell wall degrading polygalacturonases: Key events in the evolution of herbivory in beetles. Insect Biochem Mol Biol.

[19] Poelchau M.F., Coates B.S., Childers C.P., Peréz De León A.A., Evans J.D., Hackett K. (2016). Agricultural applications of insect ecological genomics. Curr Opin Insect Sci.

[20] Shelomi M., Wipfler B., Zhou X., Pauchet Y. (2020). Multifunctional cellulase enzymes are ancestral in Polyneoptera. Insect Mol Biol.

[21] Zilber-Rosenberg I., Rosenberg E. (2008). Role of microorganisms in the evolution of animals and plants: The hologenome theory of evolution. FEMS Microbiol Rev.

[22] Brune A., Dietrich C. (2015). The Gut Microbiota of Termites: Digesting the Diversity in the Light of Ecology and Evolution. Annu Rev Microbiol.

[23] Marynowska M., Sillam-Dussès D., Untereiner B., Klimek D., Goux X., Gawron P. (2023). A holobiont approach towards polysaccharide degradation by the highly compartmentalised gut system of the soil-feeding higher termite *Labiotermes labralis*. BMC Genom.

[24] Aulitto M., Martinez-Alvarez L., Fiorentino G., Limauro D., Peng X., Contursi P. (2022). A comparative analysis of *Weizmannia coagulans* genomes unravels the genetic potential for biotechnological applications. Int J Mol Sci.

[25] Sardar P., Šustr V., Chroňáková A., Lorenc F. (2022). Metatranscriptomic holobiont analysis of carbohydrate-active enzymes in the millipede *Telodeinopus aout*ii (Diplopoda, Spirostreptida). Front Ecol Evol.

[26] Grell M.N., Linde T., Nygaard S., Nielsen K.L., Boomsma J.J., Lange L. (2013). The fungal symbiont of Acromyrmex leaf-cutting ants expresses the full spectrum of genes to degrade cellulose and other plant cell wall polysaccharides. BMC Genom.

[27] Conlon B.H., O’Tuama D., Michelsen A., Crumière A.J.J., Shik J.Z. (2022). A fungal symbiont converts provisioned cellulose into edible yield for its leafcutter ant farmers. Biol Lett.

[28] Yu F., Song J., Liang J., Wang S., Lu J. (2020). Whole genome sequencing and genome annotation of the wild edible mushroom, *Russula griseocarnosa*. Genomics.

[29] Takihara H., Miura N., Aoki-Kinoshita K.F., Okuda S. (2021). Functional glyco-metagenomics elucidates the role of glycan-related genes in environments. BMC Bioinforma.

[30] Albesa-Jové D., Guerin M.E. (2016). The conformational plasticity of glycosyltransferases. Curr Opin Struct Biol.

[31] Lairson L.L., Henrissat B., Davies G.J., Withers S.G. (2008). Glycosyl transferases: Structures, functions, and mechanisms. Annu Rev Biochem.

[32] Chang T.C., Stergiopoulos I. (2015). Inter- and intra-domain horizontal gene transfer, gain-loss asymmetry and positive selection mark the evolutionary history of the CBM14 family. FEBS J.

[33] He S., Chakraborty A., Li F., Zhou C., Zhang B., Chen B. (2023). Genome-wide identification reveals conserved carbohydrate-active enzyme repertoire in termites. Front Glob Change.

[34] Sützl L., Laurent C.V.F.P., Abrera A.T., Schütz G., Ludwig R., Haltrich D. (2018). Multiplicity of enzymatic functions in the CAZy AA3 family. Appl Microbiol Biotechnol.

[35] Bredon M., Herran B., Lheraud B., Bertaux J., Grève P., Moumen B. (2019). Lignocellulose degradation in isopods: New insights into the adaptation to terrestrial life. BMC Genom.

[36] Chen W., Jiang X., Yang Q. (2020). Glycoside hydrolase family 18 chitinases: The known and the unknown. Biotechnol Adv.

[37] Franco Cairo J.P.L., Carazzolle M.F., Leonardo F.C., Mofatto L.S., Brenelli L.B., Gonçalves T.A. (2016). Expanding the knowledge on lignocellulolytic and redox enzymes of worker and soldier castes from the lower termite *Coptotermes gestroi*. Front Microbiol.

[38] Truman J.W. (2019). The evolution of insect metamorphosis. Curr Biol.

[39] Prokop J., Pecharová M., Nel A., Hörnschemeyer T., Krzemińska E., Krzemiński W. (2017). Paleozoic Nymphal Wing Pads Support Dual Model of Insect Wing Origins. Curr Biol.

[40] Sukharnikov L.O., Cantwell B.J., Podar M., Zhulin I.B. (2011). Cellulases: Ambiguous nonhomologous enzymes in a genomic perspective. Trends Biotechnol.

[41] Ruiz-Dueñas F.J., Barrasa J.M., Sánchez-García M., Camarero S., Miyauchi S., Serrano A. (2021). Genomic Analysis Enlightens Agaricales Lifestyle Evolution and Increasing Peroxidase Diversity. Mol Biol Evol.

[42] Guignard M.S., Nichols R.A., Knell R.J., Macdonald A., Romila C.A., Trimmer M. (2016). Genome size and ploidy influence angiosperm species’ biomass under nitrogen and phosphorus limitation. N Phytol.

[43] Gregory Ryan (2004). T. Genome Size Evolution in Animals. Evol Genome.

[44] Alfsnes K., Leinaas H.P., Hessen D.O. (2017). Genome size in arthropods; different roles of phylogeny, habitat and life history in insects and crustaceans. Ecol Evol.

[45] Zuzarte-Luís V., Mota M.M. (2018). Parasite Sensing of Host Nutrients and Environmental Cues. Cell Host Microbe.

[46] Orłowska M., Muszewska A. (2022). In Silico Predictions of Ecological Plasticity Mediated by Protein Family Expansions in Early-Diverging Fungi. J Fungi.

[47] Kipping L., Jehmlich N., Moll J., Noll M., Gossner M.M., Van Den Bossche T. (2024). Enzymatic machinery of wood-inhabiting fungi that degrade temperate tree species. ISME J.

[48] Dittmer N.T., Suderman R.J., Jiang H., Zhu Y.C., Gorman M.J., Kramer K.J. (2004). Characterization of cDNAs encoding putative laccase-like multicopper oxidases and developmental expression in the tobacco hornworm, *Manduca sexta*, and the malaria mosquito, *Anopheles gambiae*. Insect Biochem Mol Biol.

[49] Asano T., Seto Y., Hashimoto K., Kurushima H. (2019). Mini-review an insect-specific system for terrestrialization: Laccase-mediated cuticle formation. Insect Biochem Mol Biol.

[50] Sun W., Shen Y.H., Yang W.J., Cao Y.F., Xiang Z.H., Zhang Z. (2012). Expansion of the silkworm GMC oxidoreductase genes is associated with immunity. Insect Biochem Mol Biol.

[51] Iida K., Cox-Foster D.L., Yang X., Ko W.Y., Cavener D.R. (2007). Expansion and evolution of insect GMC oxidoreductases. BMC Evol Biol.

[52] Blaimer B.B., Santos B.F., Cruaud A., Gates M.W., Kula R.R., Mikó I. (2023). Key innovations and the diversification of Hymenoptera. Nat Commun.

[53] Wiegmann B.M., Mitter C., Farrell B. (1993). Diversification of carnivorous parasitic insects: Extraordinary radiation or specialized dead end?. Am Nat.

[54] Qu M., Guo X., Tian S., Yang Q., Kim M., Mun S. (2022). AA15 lytic polysaccharide monooxygenase is required for efficient chitinous cuticle turnover during insect molting. Commun Biol.

[55] Cook D., Manson J.S., Gardner D.R., Welch K.D., Irwin R.E. (2013). Norditerpene alkaloid concentrations in tissues and floral rewards of larkspurs and impacts on pollinators. Biochem Syst Ecol.

[56] Gong Y., Diao Q. (2017). Current knowledge of detoxification mechanisms of xenobiotic in honey bees. Ecotoxicology.

[57] Claudianos C., Ranson H., Johnson R.M., Biswas S., Schuler M.A., Berenbaum M.R. (2006). A deficit of detoxification enzymes: Pesticide sensitivity and environmental response in the honeybee. Insect Mol Biol.

[58] Sayers E.W., Bolton E.E., Brister J.R., Canese K., Chan J., Comeau D.C. (2022). Database resources of the national center for biotechnology information. Nucleic Acids Res.

[59] Mei Y., Jing D., Tang S., Chen X., Chen H., Duanmu H. (2022). InsectBase 2.0: A comprehensive gene resource for insects. Nucleic Acids Res.

[60] Sterck L., Billiau K., Abeel T., Rouze P., Van de Peer Y. (2012). ORCAE: online resource for community annotation of eukaryotes. Nat Methods.

[61] Palmer J. Funannotate: Eukaryotic Genome Annotation Pipeline. (〈https://github.com/nextgenusfs/funannotate〉) 2020.

[62] Levasseur A., Drula E., Lombard V., Coutinho P.M., Henrissat B. (2013). Expansion of the enzymatic repertoire of the CAZy database to integrate auxiliary redox enzymes. Biotechnol Biofuels.

[63] Potter S.C., Luciani A., Eddy S.R., Park Y., Lopez R., Finn R.D. (2018). HMMER web server: 2018 update. Nucleic Acids Res.

[64] Buchfink B., Xie C., Huson D.H. (2014). Fast and sensitive protein alignment using DIAMOND. Nat Methods.

[65] Zhang H., Yohe T., Huang L., Entwistle S., Wu P., Yang Z. (2018). DbCAN2: A meta server for automated carbohydrate-active enzyme annotation. Nucleic Acids Res.

[66] Bolger A.M., Lohse M., Usadel B. (2014). Trimmomatic: a flexible trimmer for Illumina sequence data. Bioinformatics.

[67] Nishimura D. (2000). RepeatMasker. Biotech Softw Internet Rep.

[68] The Galaxy platform for accessible, reproducible, and collaborative data analyses: 2024 update. Nucleic Acids Res 2024:gkae410. 10.1093/nar/gkae410 PMC11223835 38769056

[69] Hoff K.J., Stanke M. (2012). Traina - a Web Serv Appl Parameter Train Gene Predict Eukaryotes.

[70] Majoros W.H., Pertea M., Salzberg S.L. (2004). TigrScan and GlimmerHMM: Two open source ab initio eukaryotic gene-finders. Bioinformatics.

[71] Johnson A.D., Handsaker R.E., Pulit S.L., Nizzari M.M., O’Donnell C.J., De Bakker P.I.W. (2008). SNAP: A web-based tool for identification and annotation of proxy SNPs using HapMap. Bioinformatics.

[72] Manni M., Simao F.A., Robertson H.M., Gabaglio M.A., Waterhouse R.M., Misof B. (2019). The Genome of the Blind Soil-Dwelling and Ancestrally Wingless Dipluran *Campodea augens*: A Key Reference Hexapod for Studying the Emergence of Insect Innovations. Genome Biol Evol.

[73] Chamberlain S., Szoecs E., Foster Z., Arendsee Z., Boettiger C., Ram K. (2020). Taxize: Taxonomic information from around the web. R Package Version 0 9.

[74] Integrated Taxonomic Information System (ITIS) 2023. https://doi.org/https://doi.org/10.5066/F7KH0KBK.

[75] Ventura T., Palero F., Rotllant G., Fitzgibbon Q.P. (2018). Crustacean metamorphosis: an omics perspective. Hydrobiologia.

[76] Peña A., Babiker R., Chaduli D., Lipzen A., Wang M., Chovatia M. (2021). A multiomic approach to understand how *Pleurotus eryngii* transforms non-woody lignocellulosic material. J Fungi.

[77] De Bie T., Cristianini N., Demuth J.P., Hahn M.W. (2006). CAFE: A computational tool for the study of gene family evolution. Bioinformatics.

[78] Thomas G.W.C., Dohmen E., Hughes D.S.T., Murali S.C., Poelchau M., Glastad K. (2020). Gene content evolution in the arthropods. Genome Biol.

[79] Becht E., McInnes L., Healy J., Dutertre C.-A., Kwok I.W.H., Ng L.G. (2019). Dimensionality reduction for visualizing single-cell data using UMAP. Nat Biotechnol.

[80] Suhaeri M.E., Alimudin, Javaid A., Tahir Ismail M., Majahar Ali M.K. (2021). Evaluation of clustering approach with euclidean and manhattan dstance ior outlier detection. AIP Conf Proc.

[81] Murtagh F., Legendre P. (2014). Ward’s Hierarchical Agglomerative Clustering Method: Which Algorithms Implement Ward’s Criterion?. J Cl.

[82] Field A., Miles J., Field Z. (2012). Discovering Statistics Using R..

[83] Core Team R. (2021). R: A Lang Environ Stat Comput.

