# Supplementary material for: Comparative genomics reveals carbohydrate enzymatic fluctuations and herbivorous adaptations in arthropods
Source: Comput Struct Biotechnol J. 2024 Oct 18;23:3744–58. doi: 10.1016/j.csbj.2024.10.027 (PMC11543626; doi:10.1016/j.csbj.2024.10.027)
Supplement: Supplementary file 1 — Supplementary material [file mmc1.pptx]

## Slide 1
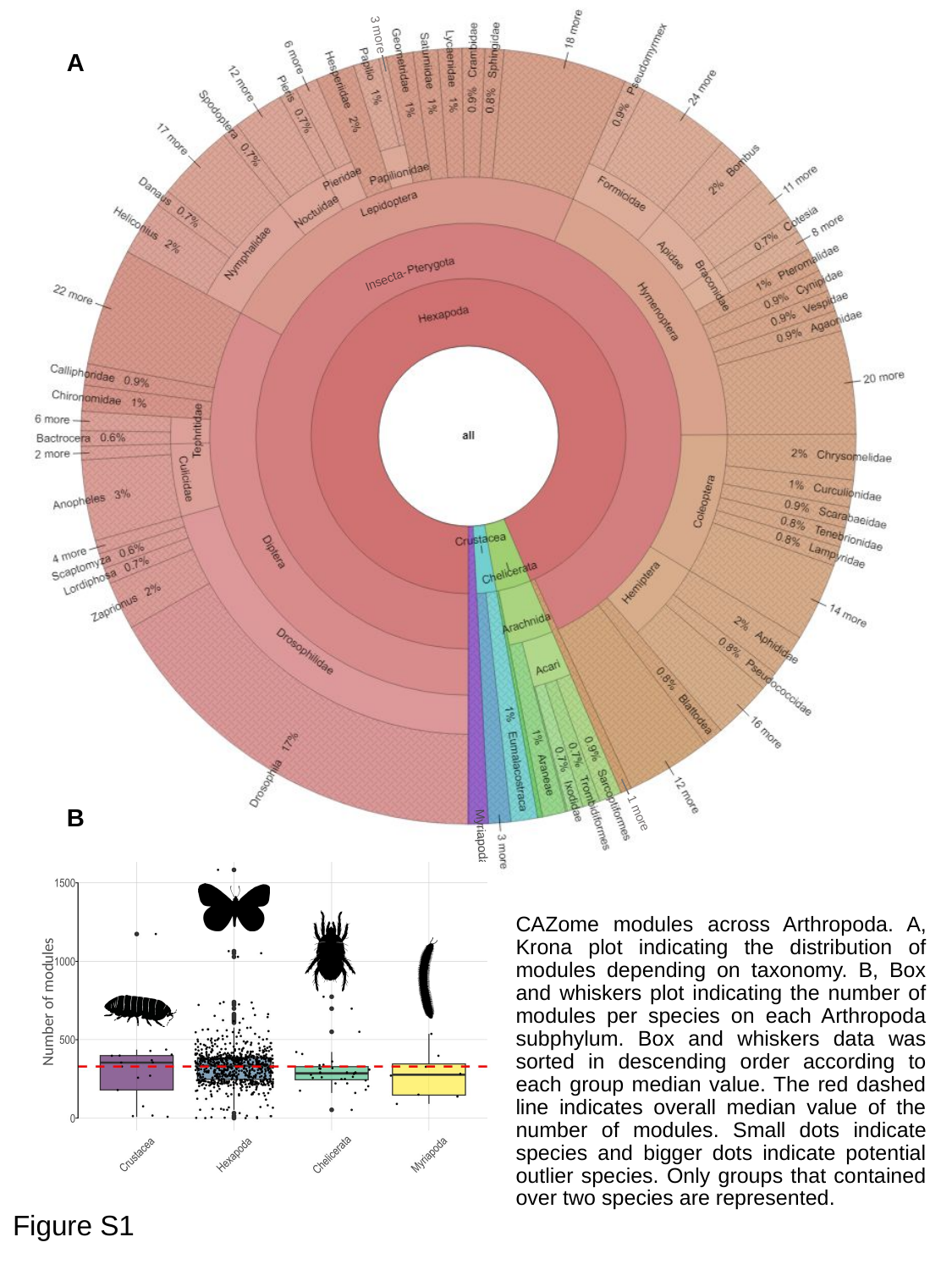

3 more
Insecta-
1 more
Myriapoda
A
B
Number of modules
CAZome modules across Arthropoda. A, Krona plot indicating the distribution of modules depending on taxonomy. B, Box and whiskers plot indicating the number of modules per species on each Arthropoda subphylum. Box and whiskers data was sorted in descending order according to each group median value. The red dashed line indicates overall median value of the number of modules. Small dots indicate species and bigger dots indicate potential outlier species. Only groups that contained over two species are represented.
# Figure S1

## Slide 2
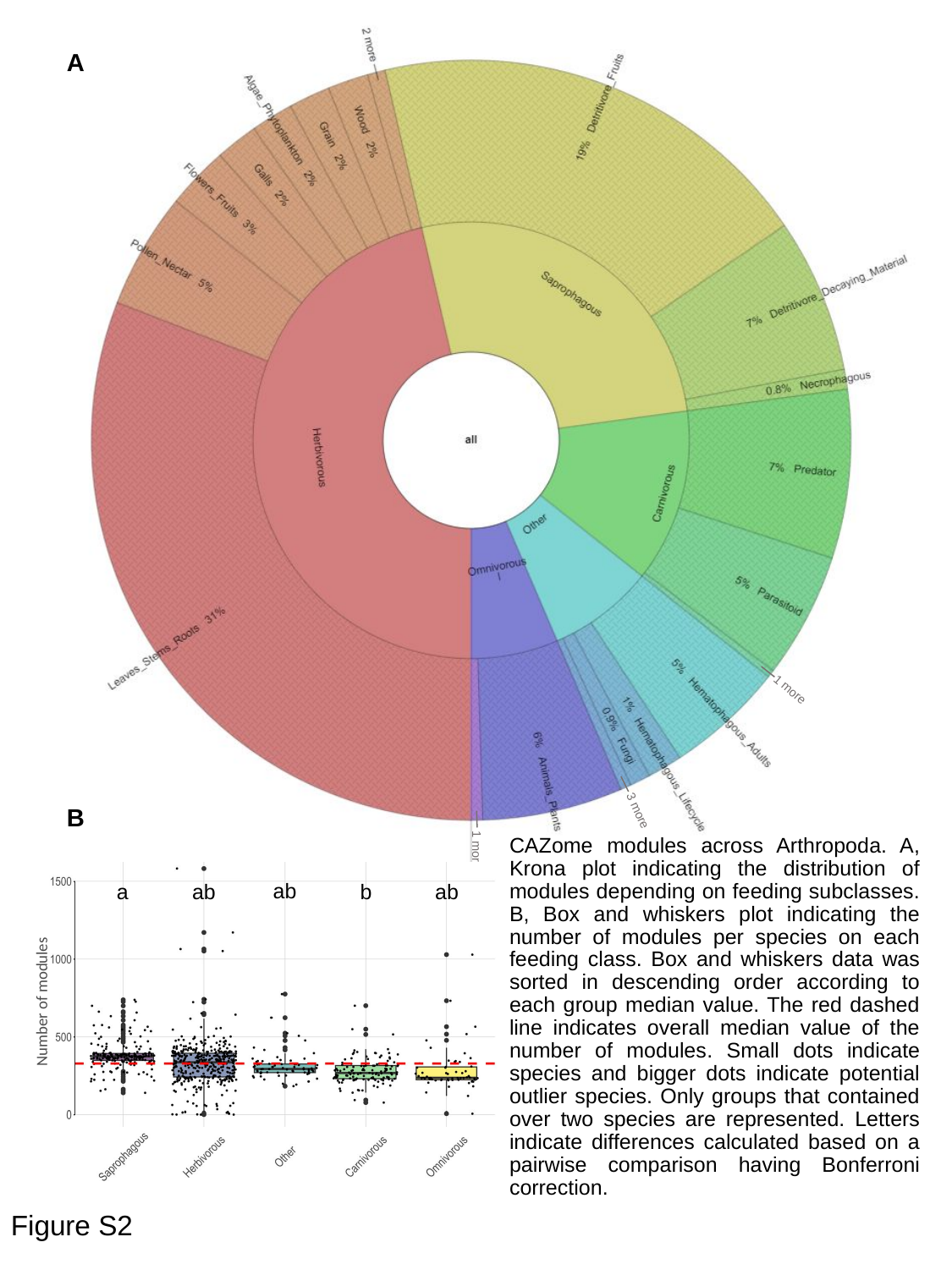

1 more
3 more
1 more
A
B
CAZome modules across Arthropoda. A, Krona plot indicating the distribution of modules depending on feeding subclasses. B, Box and whiskers plot indicating the number of modules per species on each feeding class. Box and whiskers data was sorted in descending order according to each group median value. The red dashed line indicates overall median value of the number of modules. Small dots indicate species and bigger dots indicate potential outlier species. Only groups that contained over two species are represented. Letters indicate differences calculated based on a pairwise comparison having Bonferroni correction.
Number of modules
ab
ab
a
ab
b
# Figure S2

## Slide 3
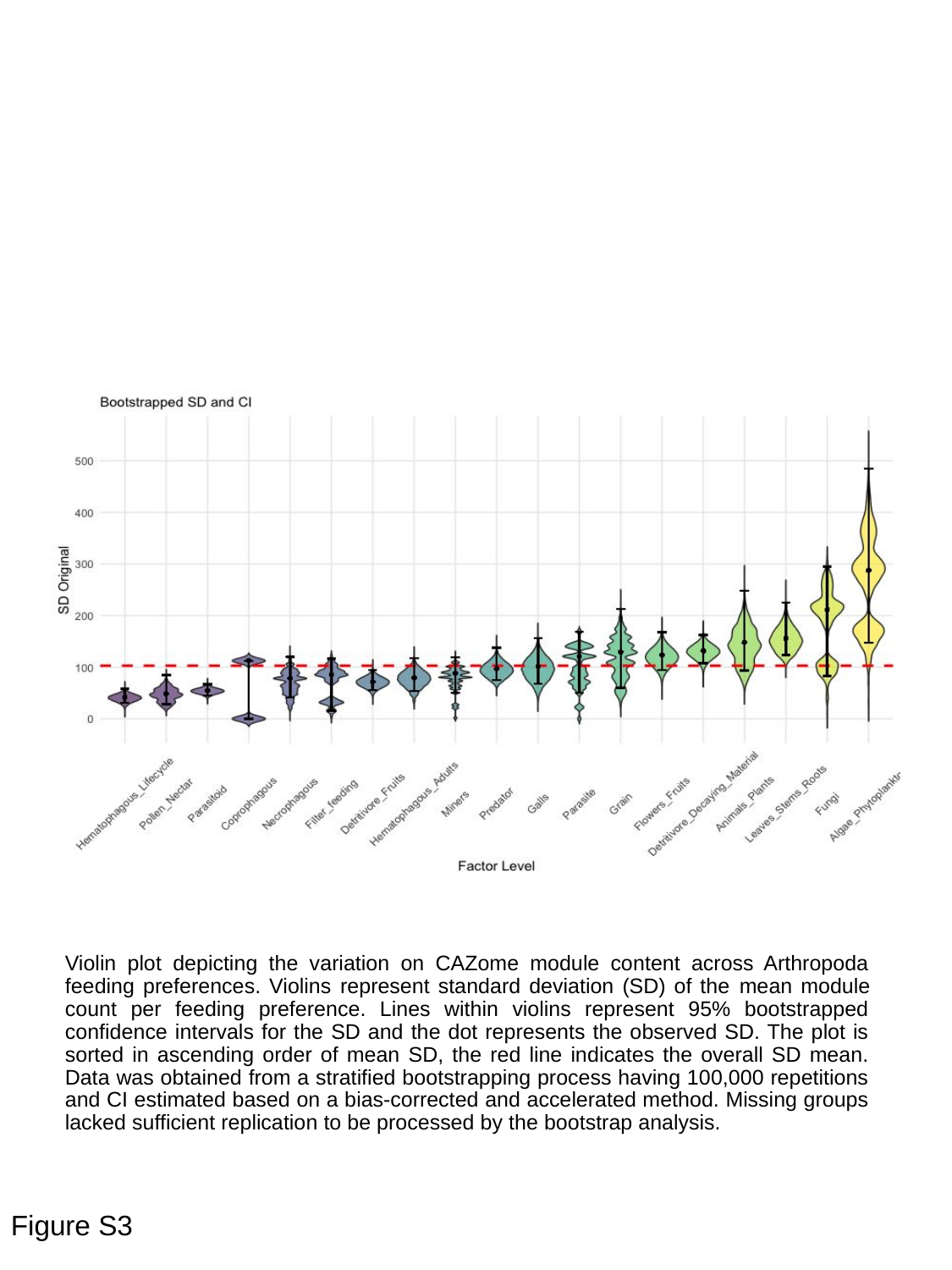

Violin plot depicting the variation on CAZome module content across Arthropoda feeding preferences. Violins represent standard deviation (SD) of the mean module count per feeding preference. Lines within violins represent 95% bootstrapped confidence intervals for the SD and the dot represents the observed SD. The plot is sorted in ascending order of mean SD, the red line indicates the overall SD mean. Data was obtained from a stratified bootstrapping process having 100,000 repetitions and CI estimated based on a bias-corrected and accelerated method. Missing groups lacked sufficient replication to be processed by the bootstrap analysis.
# Figure S3

## Slide 4
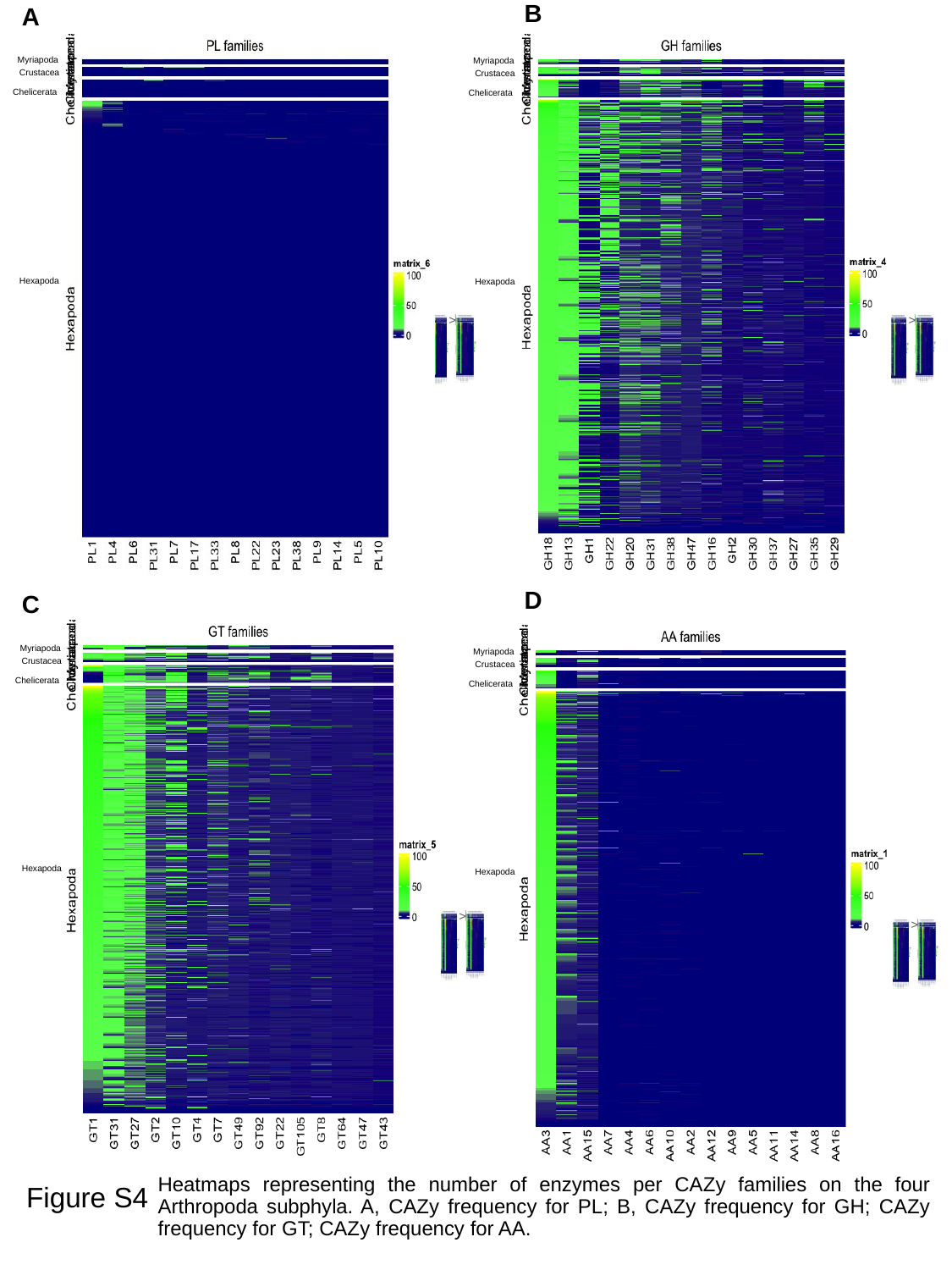

B
A
Myriapoda
Myriapoda
Crustacea
Crustacea
Chelicerata
Chelicerata
Hexapoda
Hexapoda
>
>
D
C
Myriapoda
Myriapoda
Crustacea
Crustacea
Chelicerata
Chelicerata
Hexapoda
Hexapoda
>
>
Heatmaps representing the number of enzymes per CAZy families on the four Arthropoda subphyla. A, CAZy frequency for PL; B, CAZy frequency for GH; CAZy frequency for GT; CAZy frequency for AA.
Figure S4

## Slide 5
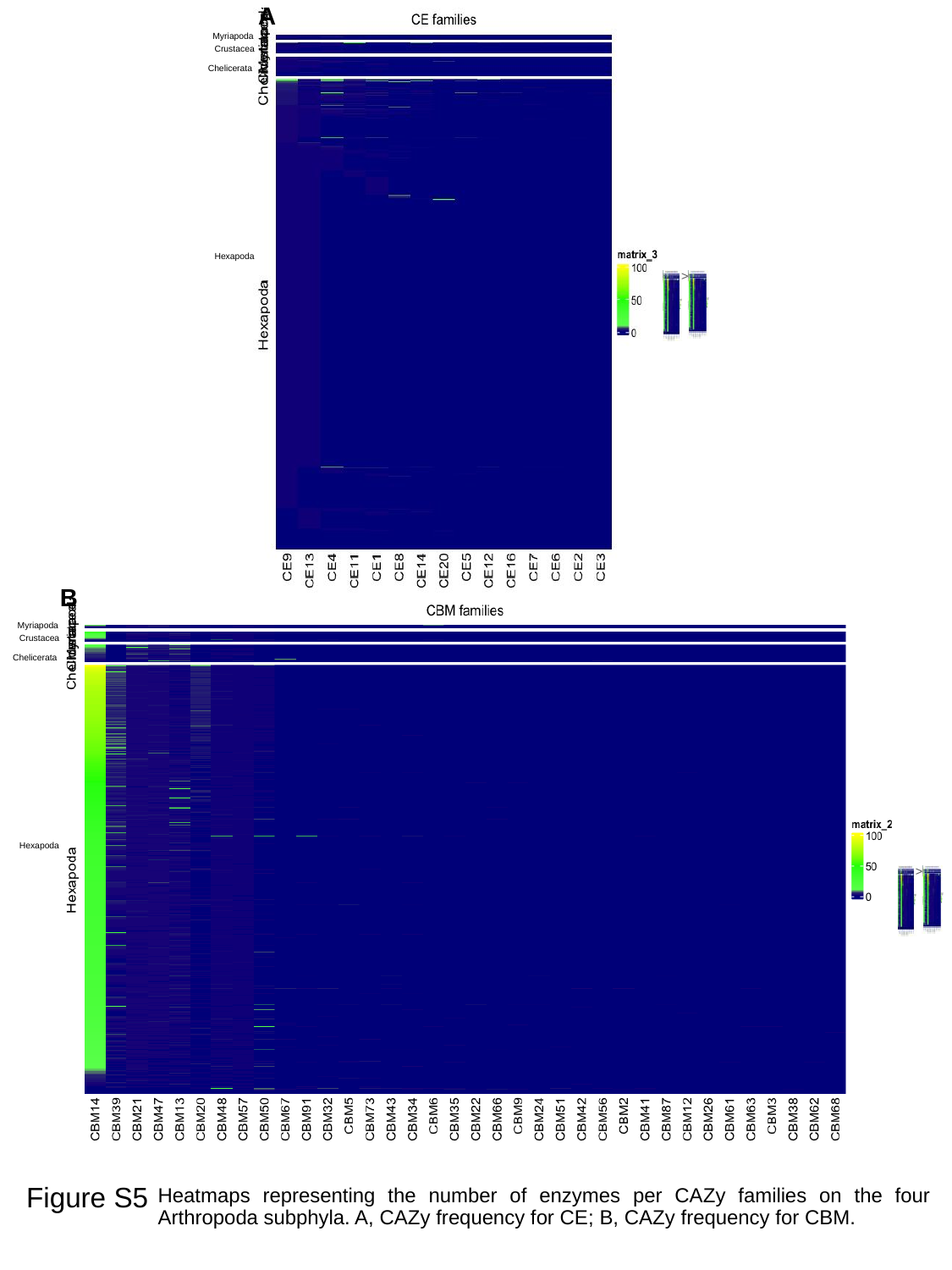

A
Myriapoda
Crustacea
Chelicerata
Hexapoda
>
B
Myriapoda
Crustacea
Chelicerata
Hexapoda
>
Heatmaps representing the number of enzymes per CAZy families on the four Arthropoda subphyla. A, CAZy frequency for CE; B, CAZy frequency for CBM.
Figure S5

## Slide 6
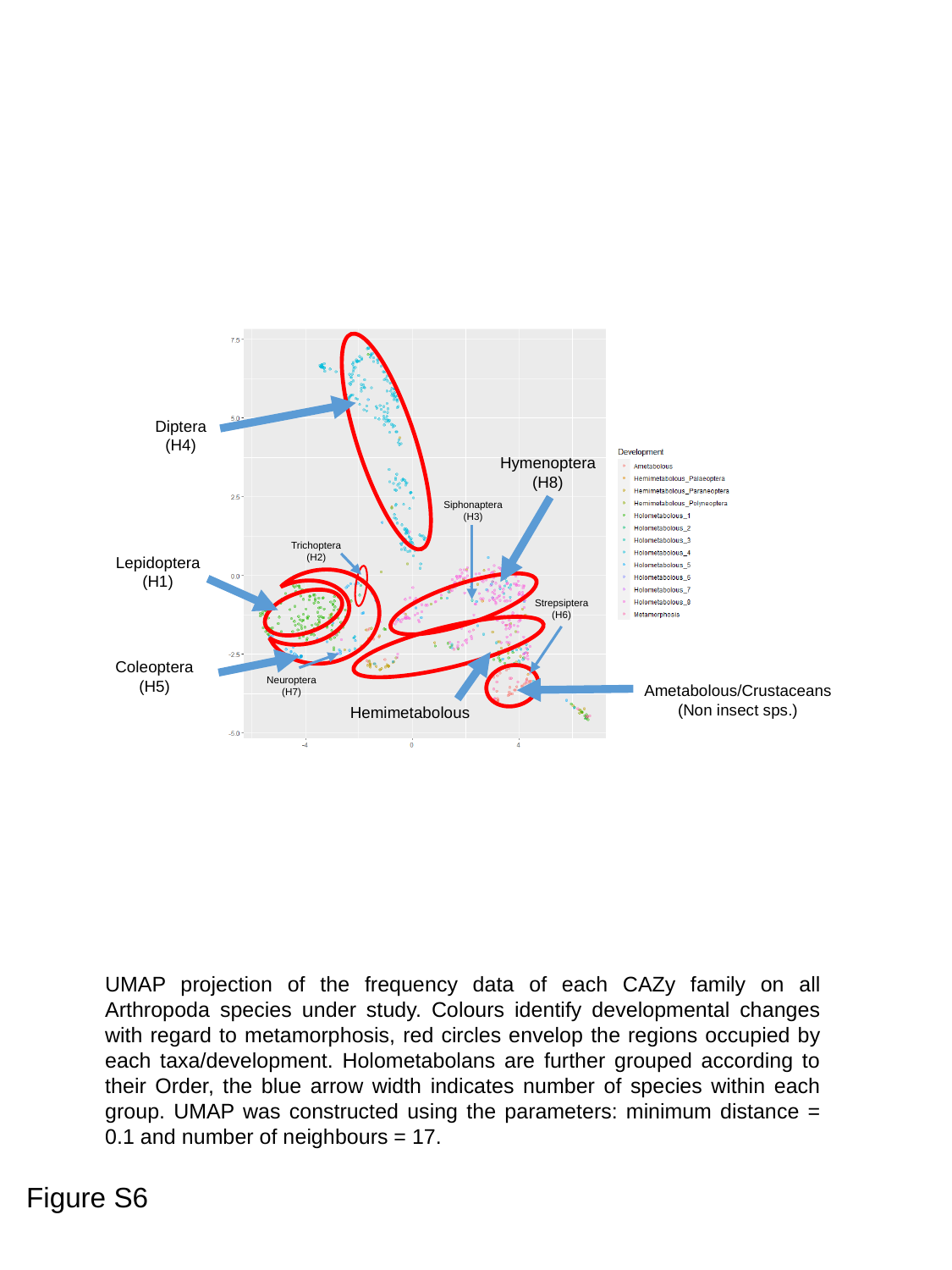

Diptera (H4)
Hymenoptera (H8)
Siphonaptera (H3)
Trichoptera (H2)
Lepidoptera (H1)
Strepsiptera (H6)
Coleoptera (H5)
Neuroptera (H7)
Ametabolous/Crustaceans
(Non insect sps.)
Hemimetabolous
UMAP projection of the frequency data of each CAZy family on all Arthropoda species under study. Colours identify developmental changes with regard to metamorphosis, red circles envelop the regions occupied by each taxa/development. Holometabolans are further grouped according to their Order, the blue arrow width indicates number of species within each group. UMAP was constructed using the parameters: minimum distance = 0.1 and number of neighbours = 17.
Figure S6

## Slide 7
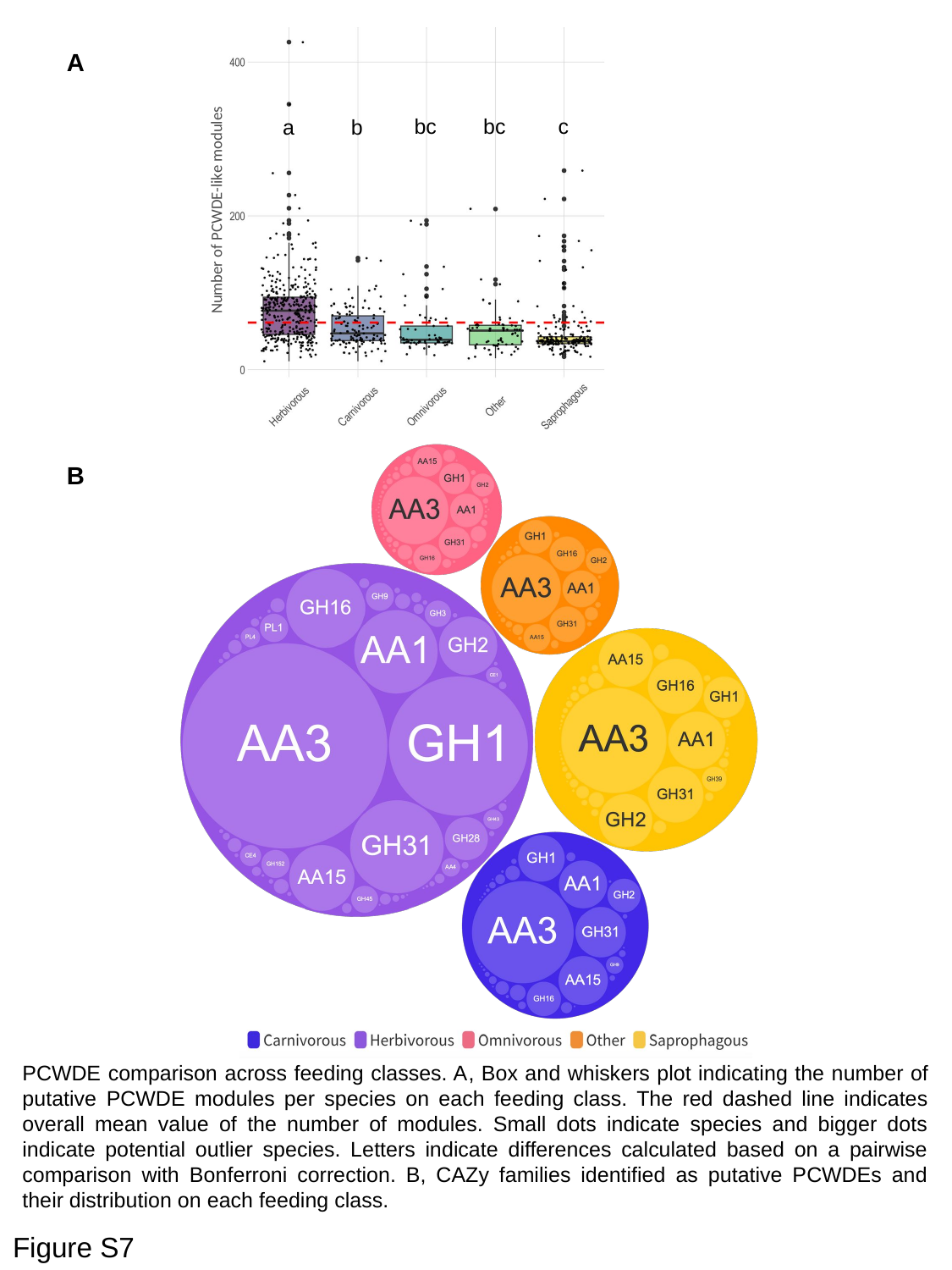

bc
c
bc
a
b
Number of PCWDE-like modules
A
B
PCWDE comparison across feeding classes. A, Box and whiskers plot indicating the number of putative PCWDE modules per species on each feeding class. The red dashed line indicates overall mean value of the number of modules. Small dots indicate species and bigger dots indicate potential outlier species. Letters indicate differences calculated based on a pairwise comparison with Bonferroni correction. B, CAZy families identified as putative PCWDEs and their distribution on each feeding class.
Figure S7

## Slide 8
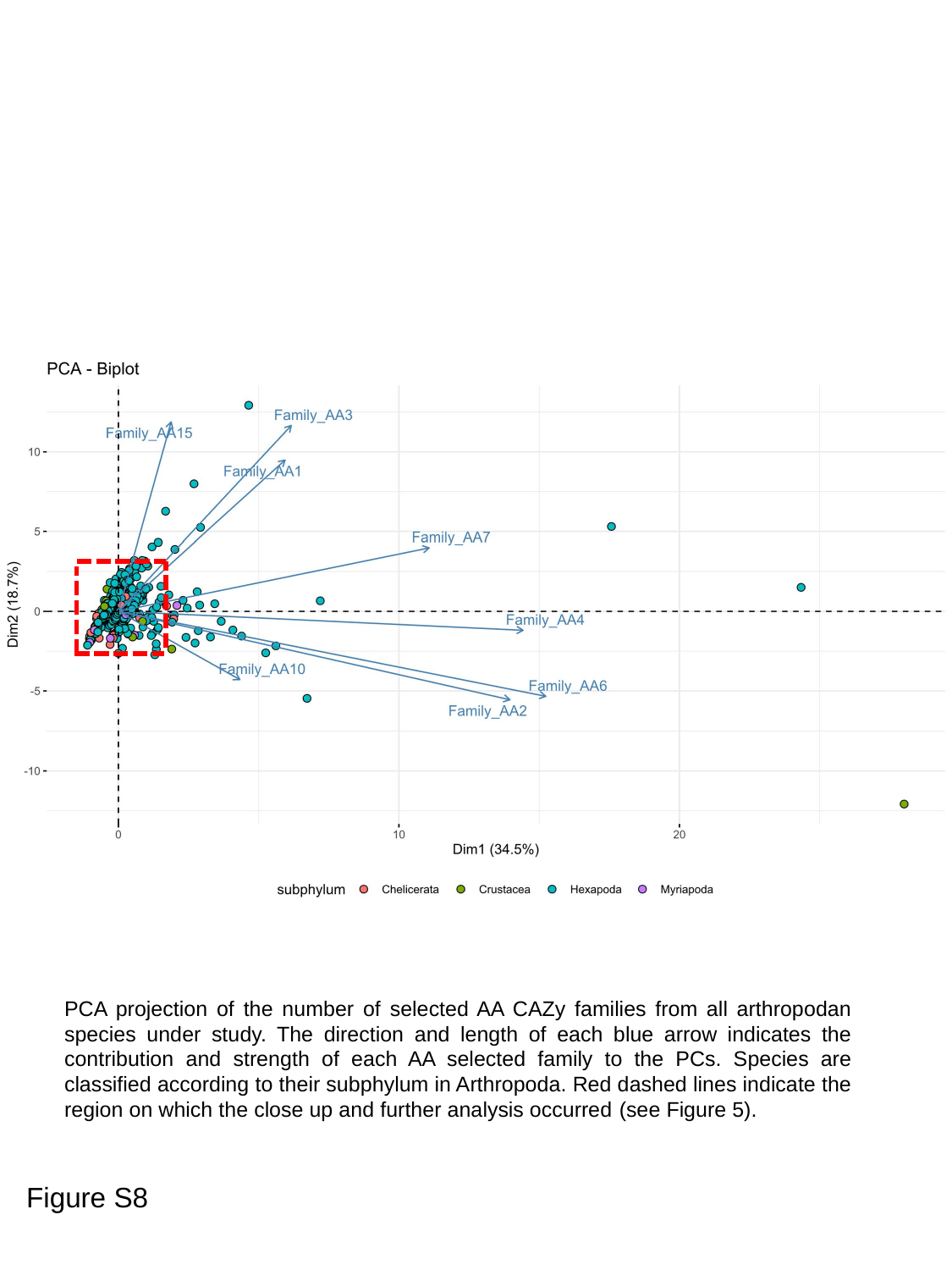

PCA projection of the number of selected AA CAZy families from all arthropodan species under study. The direction and length of each blue arrow indicates the contribution and strength of each AA selected family to the PCs. Species are classified according to their subphylum in Arthropoda. Red dashed lines indicate the region on which the close up and further analysis occurred (see Figure 5).
Figure S8

## Slide 9
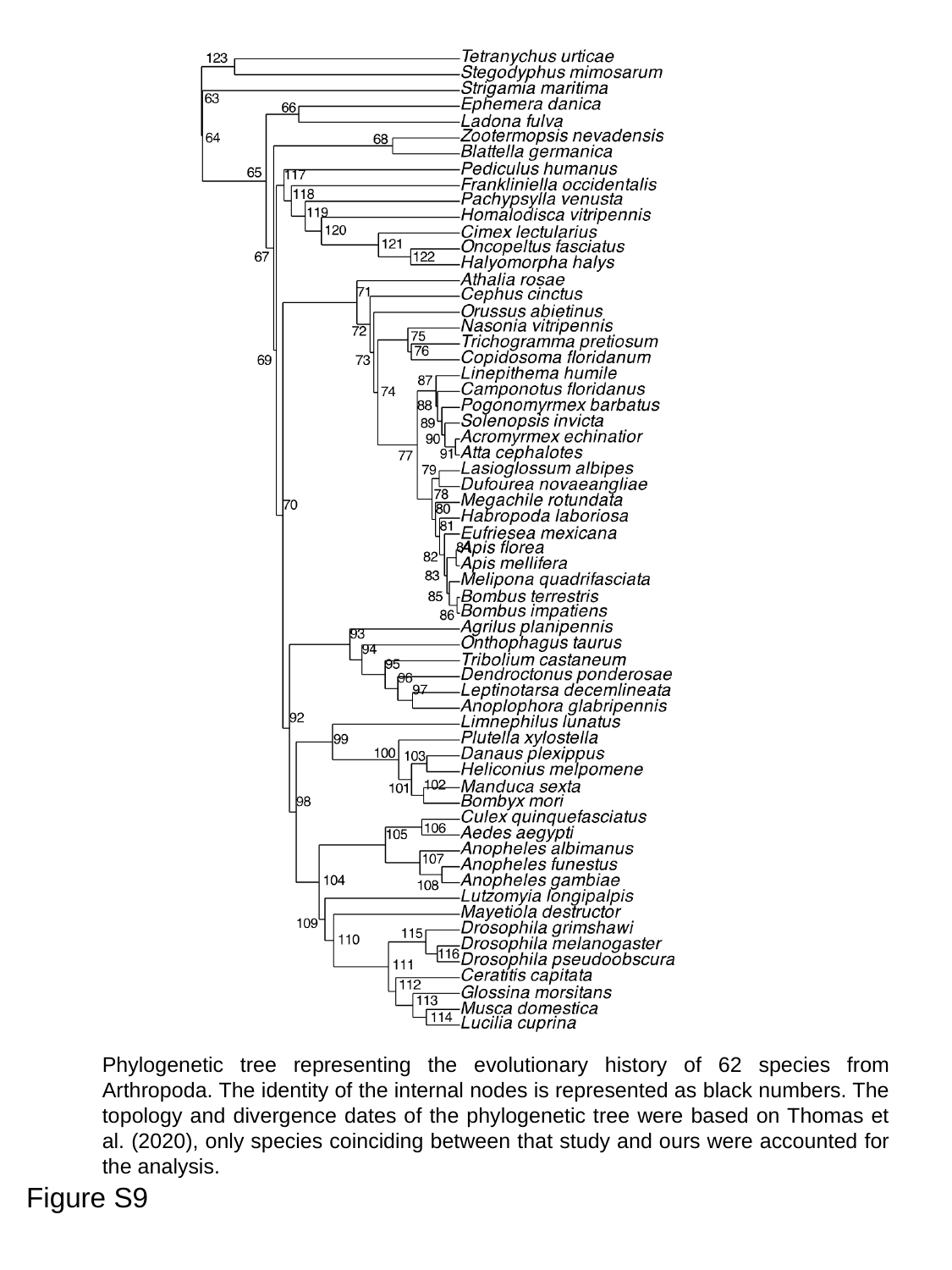

Phylogenetic tree representing the evolutionary history of 62 species from Arthropoda. The identity of the internal nodes is represented as black numbers. The topology and divergence dates of the phylogenetic tree were based on Thomas et al. (2020), only species coinciding between that study and ours were accounted for the analysis.
Figure S9
