# Supplementary material for: Comparative genomics reveals carbohydrate enzymatic fluctuations and herbivorous adaptations in arthropods
Source: Comput Struct Biotechnol J. 2024 Oct 18;23:3744–58. doi: 10.1016/j.csbj.2024.10.027 (PMC11543626; doi:10.1016/j.csbj.2024.10.027)
Supplement: Supplementary file 2 — Supplementary material [file mmc2.pdf]

CAZome GLM Models evaluated:

- model1 : Freq_Total_Modules ~ subphylum + class + Diet_Class + Diet_Subclass
- model2 : Freq_Total_Modules ~ subphylum + (1|subphylum/class) + Diet_Class + Diet_Subclass
- model3 : Freq_Total_Modules ~ subphylum + class + Diet_Class + (1|Diet_Class/Diet_Subclass)
- model4 : Freq_Total_Modules ~ (1|subphylum/class) + (1|Diet_Class/Diet_Subclass)
- model5 : Freq_Total_Modules ~ subphylum * class * Diet_Class * Diet_Subclass
- model6 : Freq_Total_Modules ~ (1|subphylum/class) * (1|Diet_Class/Diet_Subclass)
- model7 : Freq_Total_Modules ~ class*Diet_Subclass
- model8 : Freq_Total_Modules ~ subphylum * Diet_Class
- model9 : Freq_Total_Modules ~ subphylum+Diet_Class

Model notes:
~ : Indicates dependence of the left hand variable on the right hand variables
* : Indicates interaction among factor levels
+ : Indicates the inclusion in the model as independent predictors
(1|factor1/factor2) : Indicates that factor2 levels are nested within factor1
Taxa factors: Arthropod Subphylum and Class
Dietary factors: Diet Class - carnivore, herbivore, omnivore, saprophagous and other; Diet Subclass - specific food preferences such as wood, grain, fungi, blood, pollen etc.

Model IDs:

Model ID	**Model Name**
model1	Simple
model2	Nested_Class
model3	Nested_DietSubclass
model4	Nested
model5	Simple_Int
model6	Nested_Int
model7	Class
Model8	Subp
Model9	Subp_Simple

Model evaluation criteria

	Poisson distribution			Negative binomial distribution		
**Model name**	**df**	**AIC**	**Overdispersion**	**df**	**AIC**	**Overdispersion**
**model1 (Simple)**	22	37693.77	42,32	23	9950.69	0.79
**model2**	23	37696.12	42,37	24	9952.70	0.79
**model3**	11	37771.46	41,72	12	9959.17	0.78
**model4**	5	37788.89	41,39	6	9961.81	0.74
**model5**	25	37634.44	42,45	26	9955.44	0.79
**model6**	5	37788.89	41,39	6	9961.81	0.74
**model7**	25	37634.44	42,45	26	9955.44	0.79
**Model8**	14	43375.91	48,60	15	10311.65	0.81
**Model9**	8	43672.64	48,88	9	10304.84	0.81

The model selected to compare frequency data is highlighted in red. Selection was based on a balance between overdispersion control, AIC value and model simplicity.

Model1 results

General model results

	Df	Deviance	Resid. Df	Resid. Dev	Pr(>Chi)
NULL			789	958.16	
subphylum	3	11.67	786	946.48	0.008 **
class	1	0.62	785	94.58	0.42
Diet_Class	4	50.34	781	895.52	3,06E+04 ***
Diet_Subclass	13	44.03	768	851.48	3,03E+09 ***

Post-hoc analysis (Pairwise-Bonferroni correction)

Diet_Subclass	***lsmean***	***SE***	***asymp.LCL***	***asymp.UCL***	**.group**
Pollen_Nectar	*5,33403E+14*	*1,01237E+14*	*5,03119E+14*	*5,63686E+14*	e
Parasitoid	*5,43494E+14*	*1,01491E+13*	*5,13134E+13*	*5,73853E+14*	de
Animals_Plants	*5,49501E+14*	*1,00902E+13*	*5,19318E+14*	*5,79684E+14*	cde
Predator	*5,49755E+14*	*9,14918E+14*	*5,22387E+14*	*5,77123E+14*	cde
Hematophagous_Lifecycle	*5,42309E+14*	*1,35681E+14*	*5,01722E+14*	*5,82895E+14*	bcde
Flowers_Fruits	*5,49006E+14*	*1,17677E+14*	*5,13806E+14*	*5,84207E+14*	bcde
Filter_feeding	*5,40248E+14*	*2,01988E+14*	*4,79827E+14*	*6,00669E+14*	abcde
Miners	*5,47528E+14*	*1,87463E+14*	*4,91452E+14*	*6,03604E+14*	abcde
Fungi	*5,57638E+14*	*2,01686E+14*	*4,97307E+14*	*6,17968E+14*	abcde
Galls	*5,59689E+14*	*1,35298E+14*	*5,19217E+14*	*6,00161E+14*	abcde
Algae_Phytoplankton	*5,59856E+14*	*1,65071E+14*	*5,10479E+13*	*6,09234E+14*	abcde
Necrophagous	*5,60926E+13*	*1,76285E+14*	*5,08193E+14*	*6,13658E+14*	abcde
Hematophagous_Adults	*5,60896E+14*	*1,05441E+13*	*5,29355E+14*	*5,92437E+13*	bcd
Wood	*5,79201E+14*	*1,451E+14*	*5,35797E+14*	*6,22605E+14*	abcd

Detritivore_Decaying_Material	*5,70598E+14*	*9,60723E+14*	*5,4186E+14*	*5,99336E+14*	abc
Grain	*5,81099E+13*	*1,45086E+14*	*5,37699E+14*	*6,24499E+14*	abc
Leaves_Stems_Roots	*5,68214E+13*	*8,80752E+14*	*5,41868E+14*	*5,9456E+14*	b
Detritivore_Fruits	*5,81639E+14*	*9,17673E+14*	*5,54189E+14*	*6,0909E+14*	a

Diet_Class	***lsmean***	***SE***	***asymp.LCL***	***asymp.UCL***	.group
Omnivorous	*544.874.443.835.238*	*0.127703230746677*	*511.980.271.443.722*	*577.768.616.226.754*	ab
Carnivorous	*546.624.132.086.349*	*0.0880705274314612*	*523.938.667.552.652*	*569.309.596.620.045*	a
Other	*553.614.131.192.739*	*0.109741599036592*	*525.346.568.531.062*	*581.881.693.854.416*	ab
Herbivorous	*559.749.649.474.966*	*0.0847155098255807*	*537.928.380.207.585*	*581.570.918.742.348*	ab
Saprophagous	*571.054.311.175.276*	*0.0983560675121378*	*545.719.467.087.316*	*596.389.155.263.236*	b

PCWDE GLM analysis

Model: PCWDE_total ~ Diet_Class

Model notes:
~ : Indicates dependence of the left hand variable on the right hand variables

Model results:

	Df	Deviance	Resid. Df	Resid. Dev	Pr(>Chi)
NULL			814	1002.16	
Diet_Class	4	109.8	810	892.36	2.2e-16***

PCWDE Post-hoc analysis (Pairwise-Bonferroni correction)

**Contrast**	**Estimate**	**SE**	**Z.ratio**	**p.value**
Carnivorous - Herbivorous	-0.3185	0.0585	-5.449	<.001
Carnivorous - Omnivorous	0.0469	0.0885	0.530	0.6622
Carnivorous - Other	0.0857	0.0858	0.998	0.4548
Carnivorous - Saprophagous	0.1557	0.0658	2.367	0.0359
Herbivorous - Omnivorous	0.3654	0.0779	4.693	<.001
Herbivorous - Other	0.4042	0.0749	5.398	<.001
Herbivorous - Saprophagous	0.4742	0.0507	9.362	<.001
Omnivorous - Other	0.0388	0.1001	0.387	0.6986
Omnivorous - Saprophagous	0.1088	0.0835	1.303	0.3210
Other - Saprophagous	0.0701	0.0807	0.868	0.4819

PCA analysis

Kaiser-Meyer-Olkin factor adequacy, overall: 0.69

MSA for each AA family that remained:
Family AA1: 0.61
Family AA2: 0.68
Family AA3: 0.60
Family AA4: 0.79
Family AA6: 0.66
Family AA7: 0.75
Family AA10: 0.72
Family AA15: 0.57

Bartlett's test results: X^2, 1782.015; $p < 0.001$; df, 28

Determinant value: 0.2929615

CAFE5 analysis

Models evaluated:

**Gamma** model for gene family distribution		**Base** model for gene family distribution
**Uniform** frequency distribution	**Poisson** frequency distribution	**Uniform** frequency distribution
k = 1	k = 1	
k = 2	**k = 2**	
k = 3	k = 3	
k = 4	k = 4	
k = 5	k = 5	
k = 6	k = 6	
k = 7	k = 7	
k = 8	k = 8	
k = 9	k = 9	
k = 10	k = 10	

k : Indicates the number of discrete gamma rate categories

CAZy count data was processed in parallel using 21 models: the 10 Gamma+Uniform models, the 10 Gamma+Poisson models and the Base+Uniform model. The model selected to determine enzyme family fluctuations is highlighted in red.

Selected model results

Model Gamma Final Likelihood (-lnL): 6521.44
Lambda: 0.0011926183062924
Epsilon: 0.00858916
Maximum possible lambda for this topology: 0.00175953
123 values were attempted (2% rejected)
The following families had failure rates >20% of the time:
CBM14 had 51 failures
Alpha: 1.98874

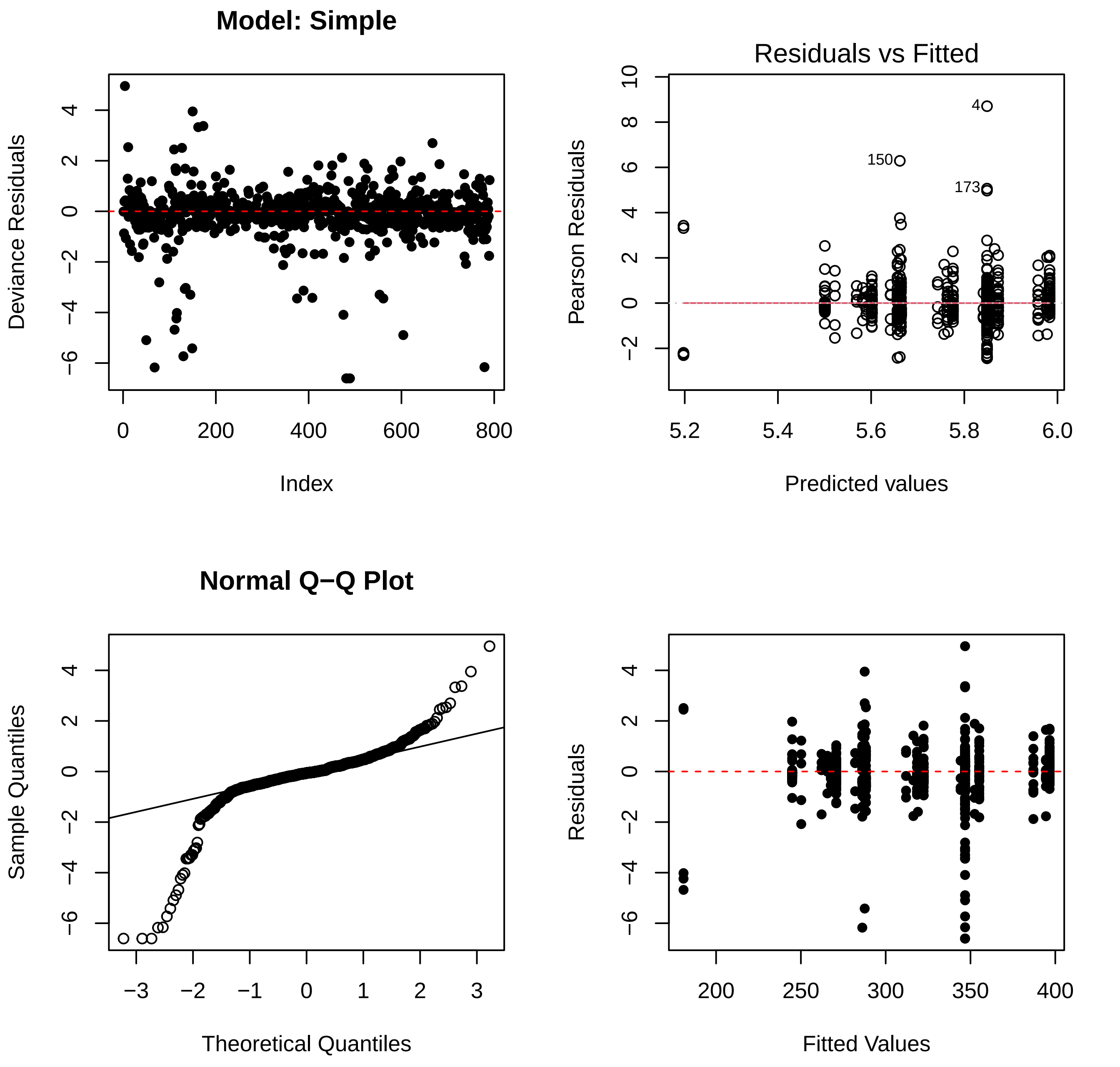
Model: Simple
Deviance Residuals
Index
Residuals vs Fitted
Pearson Residuals
Predicted values
4
150
173
Normal Q−Q Plot
Sample Quantiles
Theoretical Quantiles
Residuals
Fitted Values

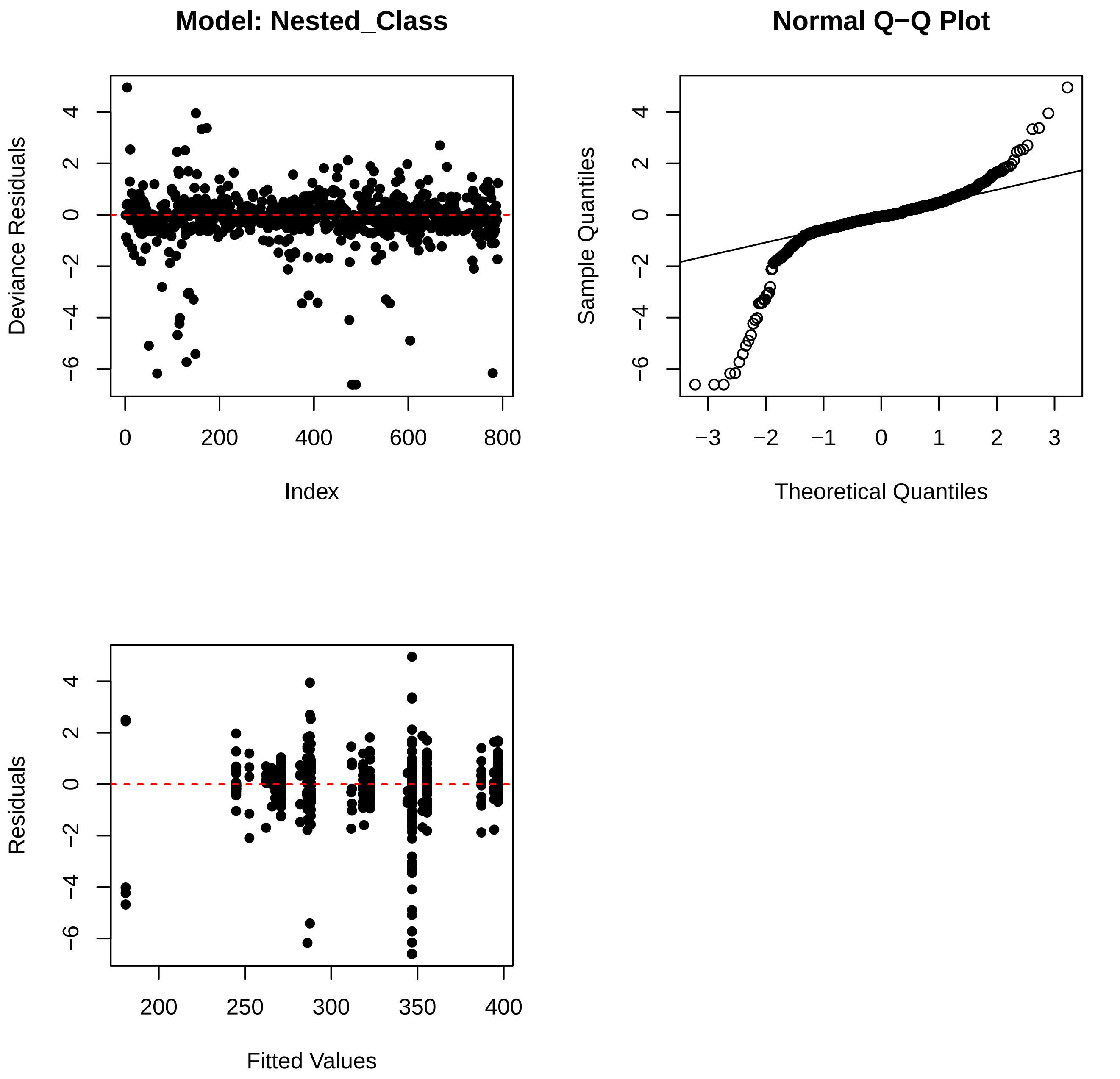
Model: Nested_Class
Deviance Residuals
Index
Normal Q−Q Plot
Sample Quantiles
Theoretical Quantiles
Residuals
Fitted Values

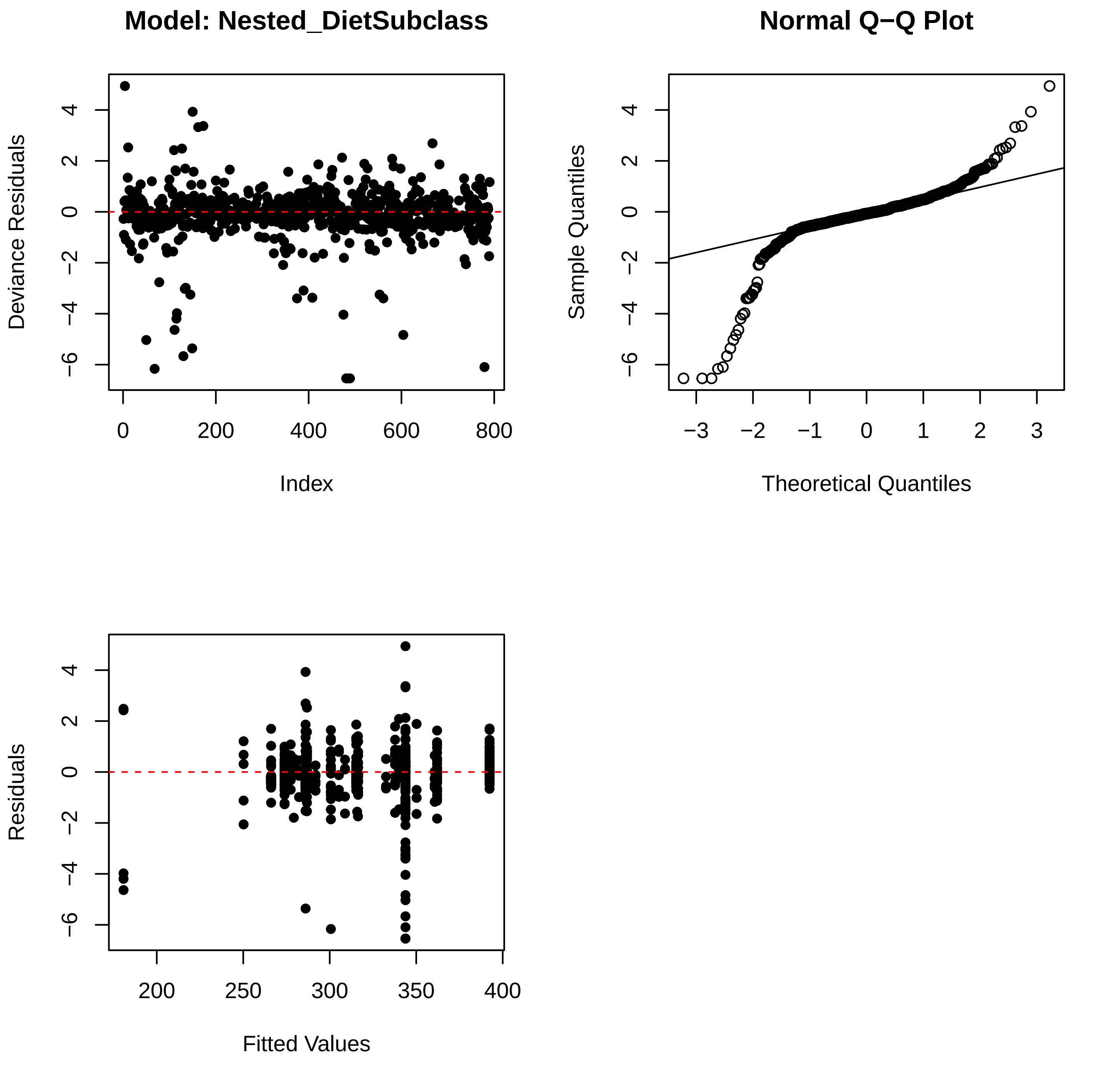

Model: Nested_DietSubclass
Deviance Residuals
Index
0
200
400
600
800
4
2
0
−2
−4
−6
Normal Q−Q Plot
Sample Quantiles
Theoretical Quantiles
−3
−2
−1
0
1
2
3
Residuals
Fitted Values
200
250
300
350
400

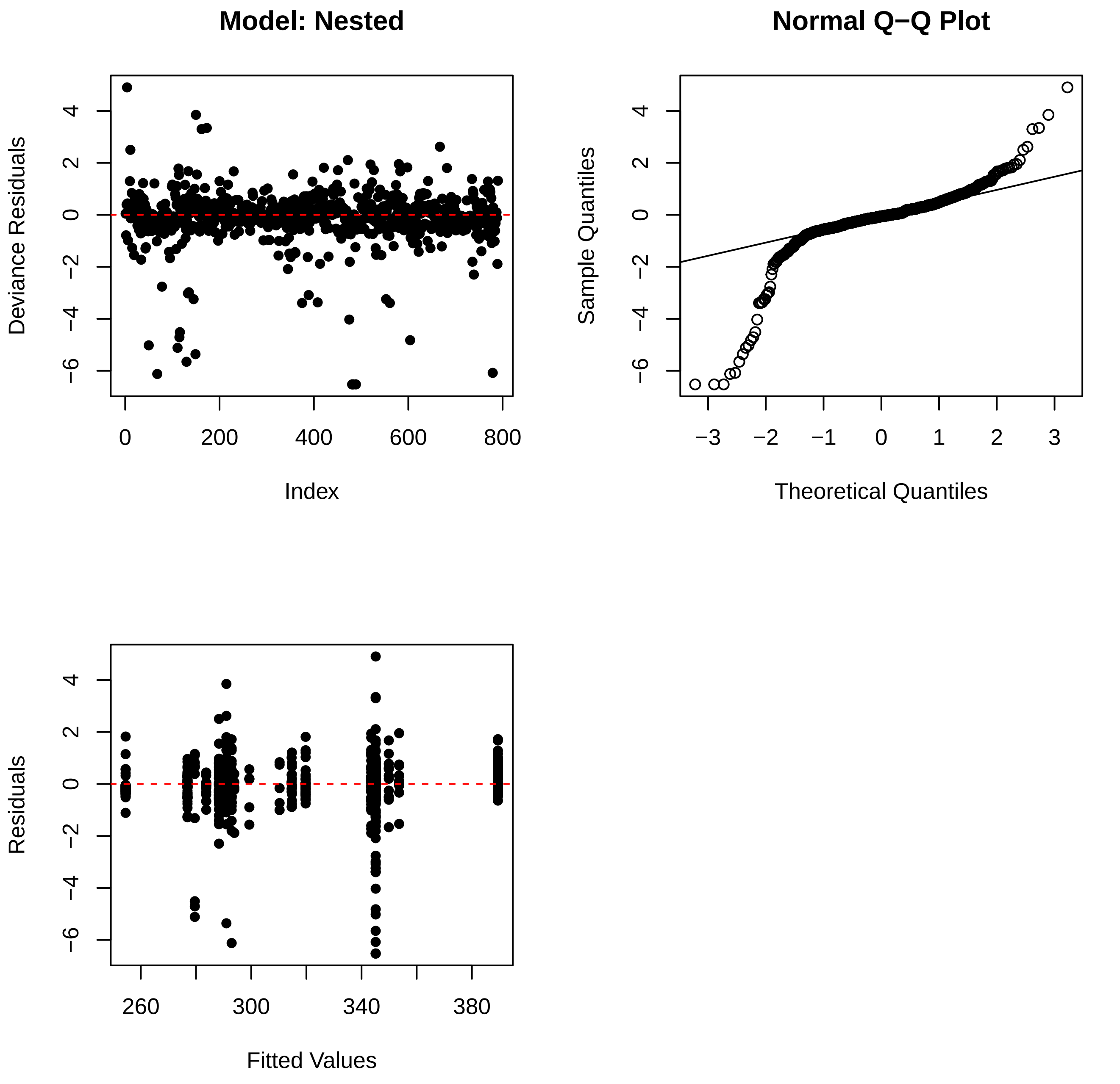
Model: Nested
Deviance Residuals
Index
Normal Q−Q Plot
Sample Quantiles
Theoretical Quantiles
Residuals
Fitted Values

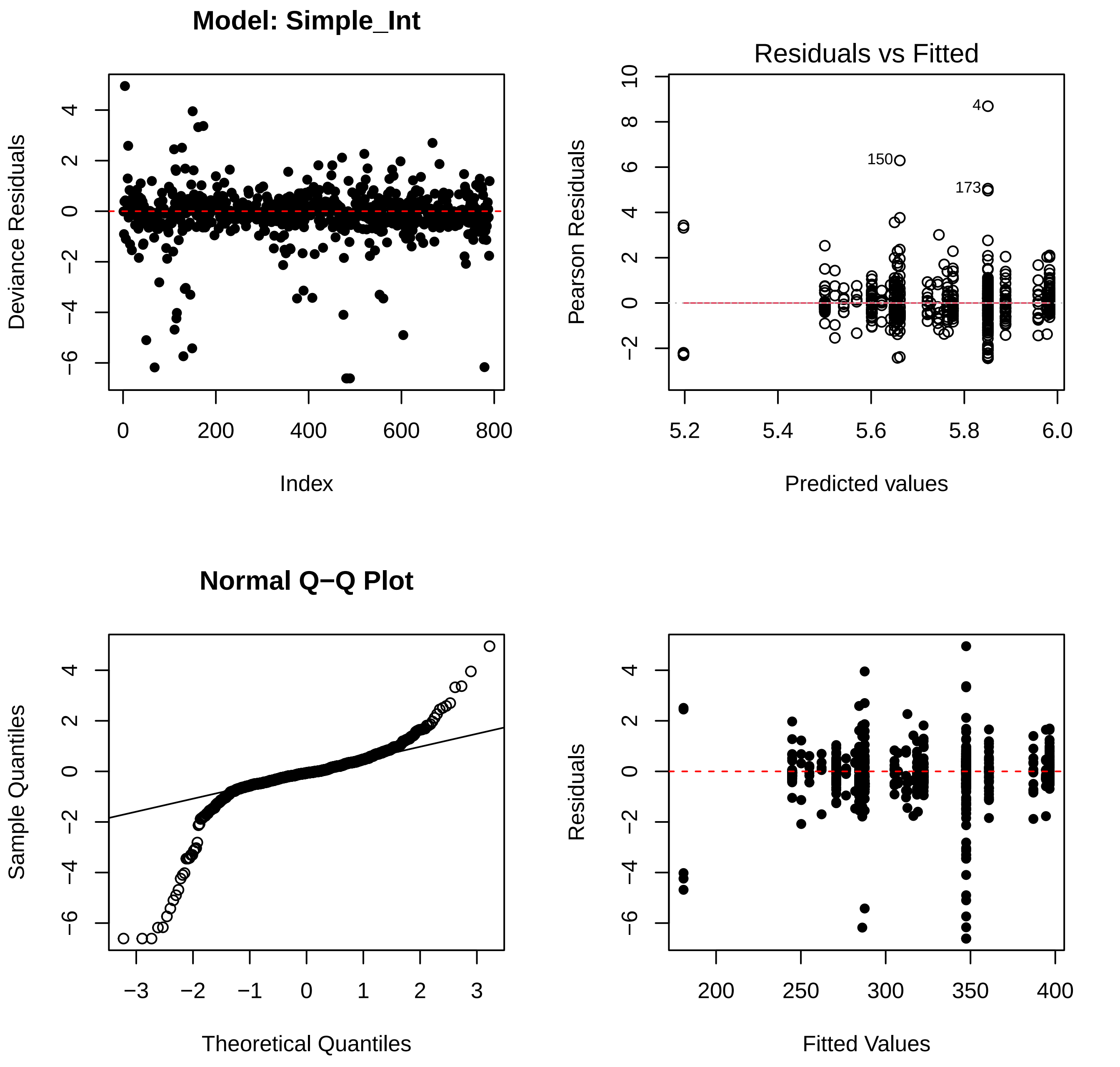

Model: Simple_Int
Deviance Residuals
Index
Residuals vs Fitted
Pearson Residuals
Predicted values
150
4
173
Normal Q−Q Plot
Sample Quantiles
Theoretical Quantiles
Residuals
Fitted Values

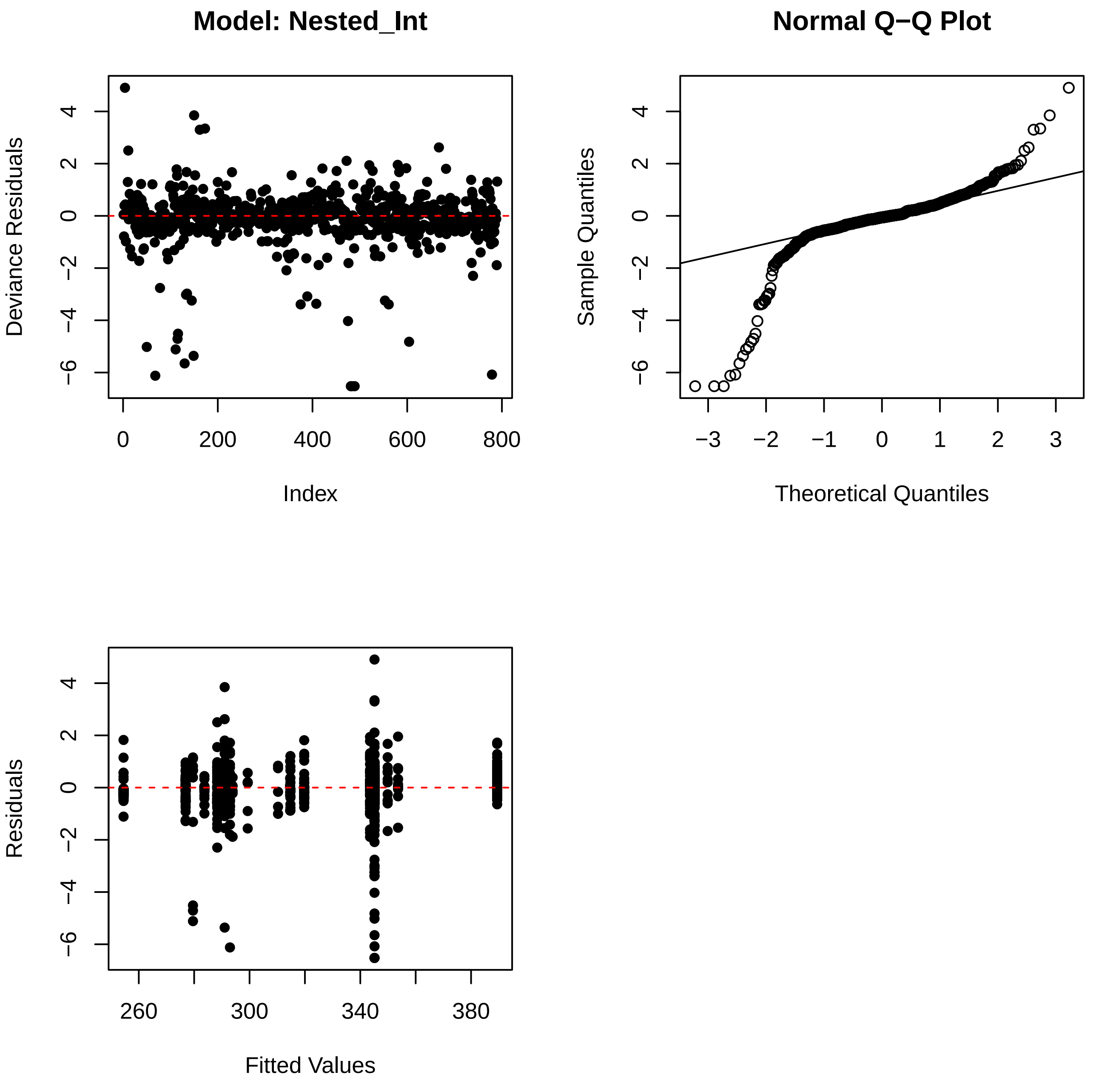
Model: Nested_Int
Deviance Residuals
Index
0
200
400
600
800
4
2
0
−2
−4
−6
Normal Q−Q Plot
Sample Quantiles
Theoretical Quantiles
−3
−2
−1
0
1
2
3
Residuals
Fitted Values
260
300
340
380

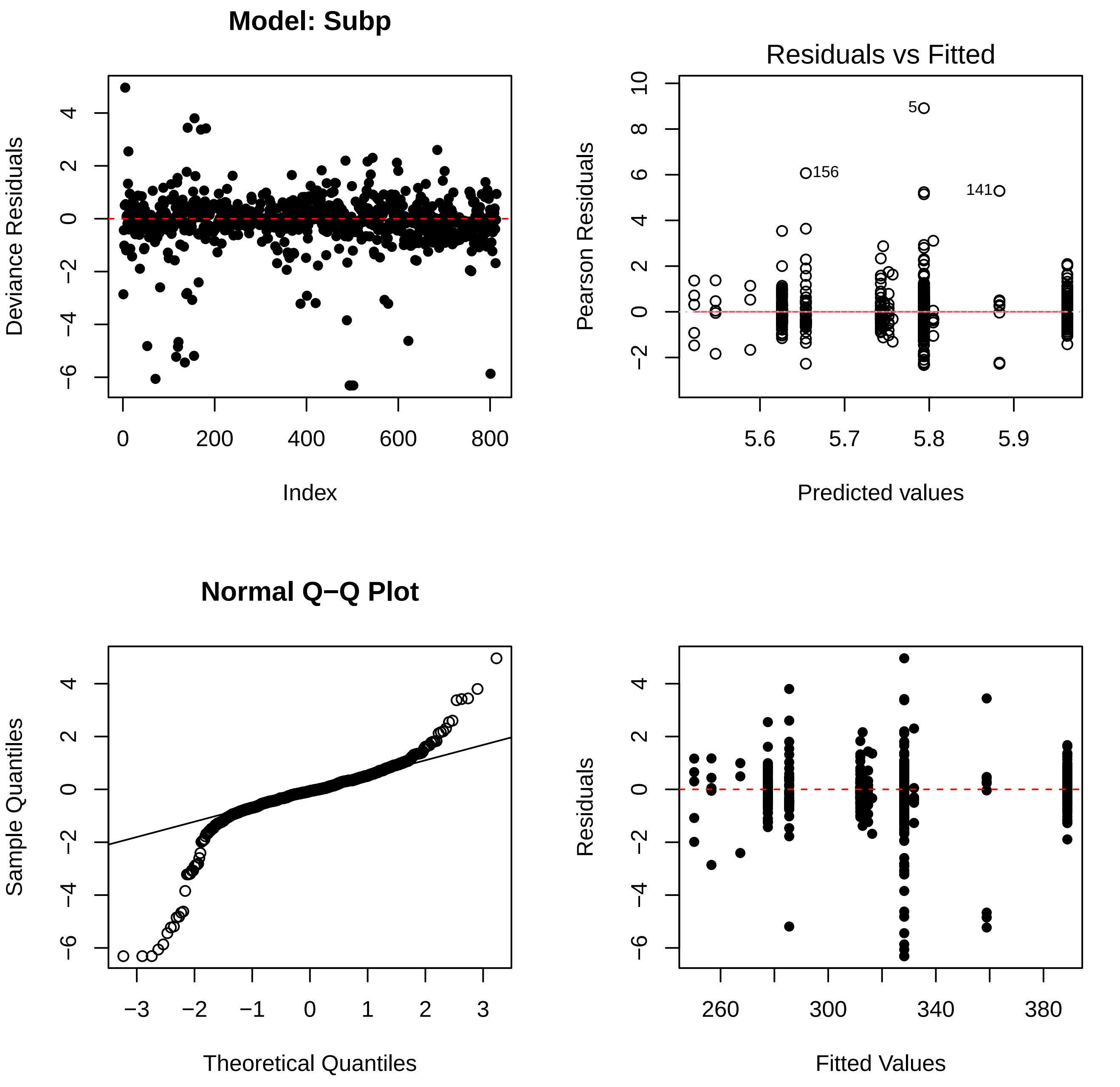
Model: Subp
Deviance Residuals
Index
Residuals vs Fitted
Pearson Residuals
Predicted values
5
156
141
Normal Q−Q Plot
Sample Quantiles
Theoretical Quantiles
Residuals
Fitted Values

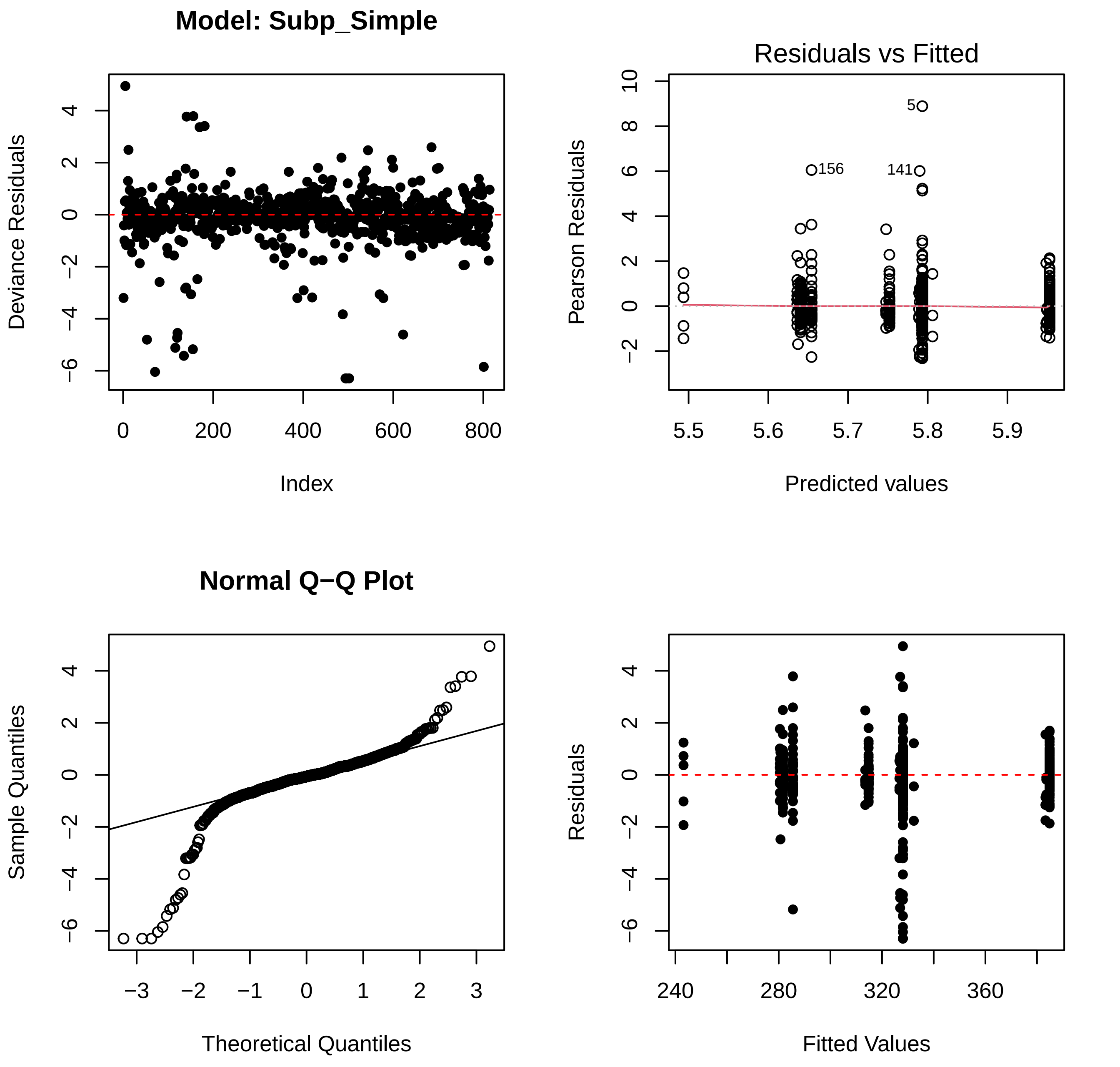

Model: Subp_Simple
Deviance Residuals
Index
Residuals vs Fitted
Pearson Residuals
Predicted values
5
156
141
Normal Q−Q Plot
Sample Quantiles
Theoretical Quantiles
Residuals
Fitted Values

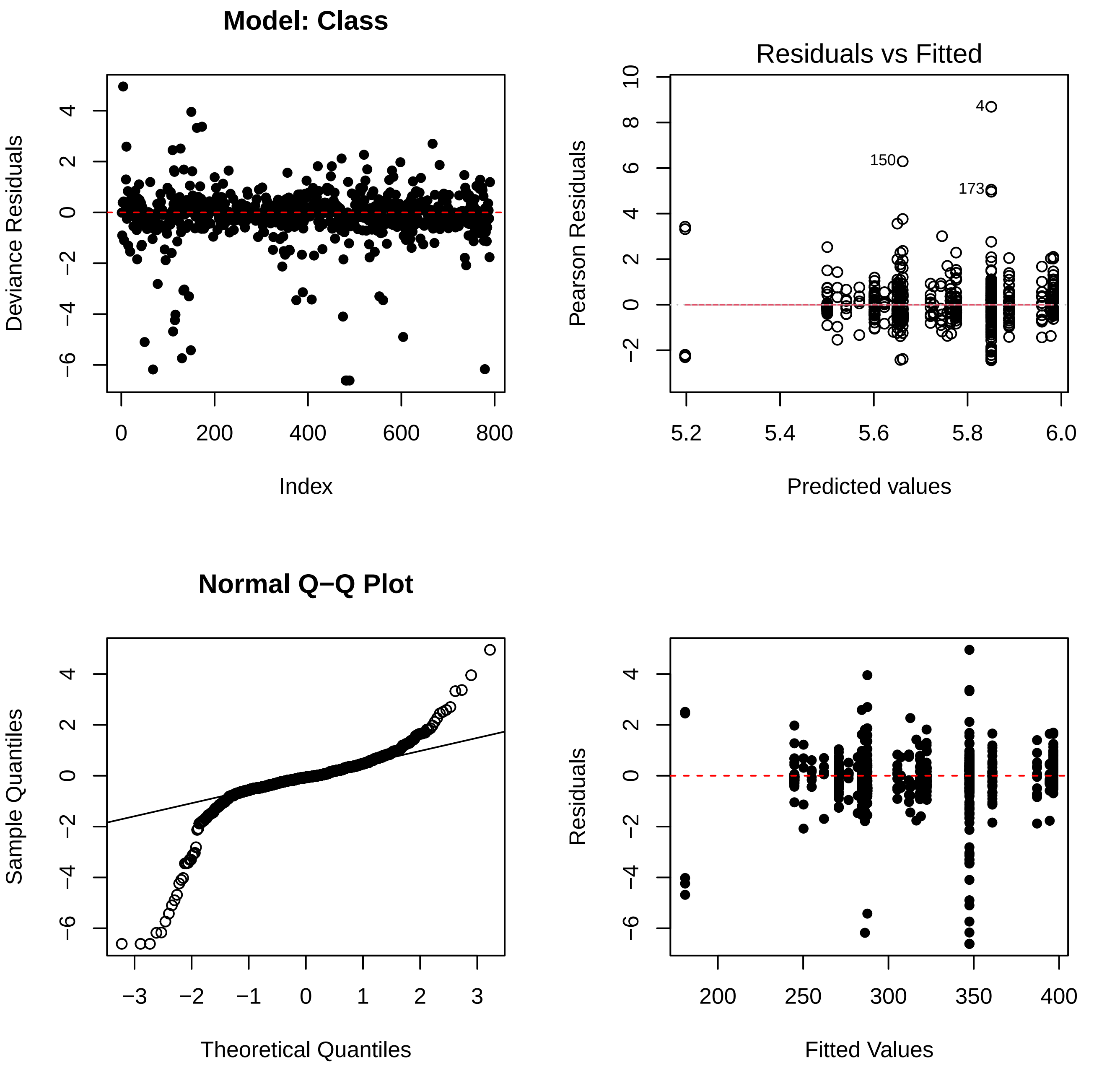
Model: Class
Deviance Residuals
Index
Residuals vs Fitted
Pearson Residuals
Predicted values
4
150
173
Normal Q−Q Plot
Sample Quantiles
Theoretical Quantiles
Residuals
Fitted Values
