# Supplementary figures and images for: Comparative genomics reveals carbohydrate enzymatic fluctuations and herbivorous adaptations in arthropods
Source: Comput Struct Biotechnol J. 2024 Oct 18;23:3744–58. doi: 10.1016/j.csbj.2024.10.027 (PMC11543626; doi:10.1016/j.csbj.2024.10.027)

## Slide 1
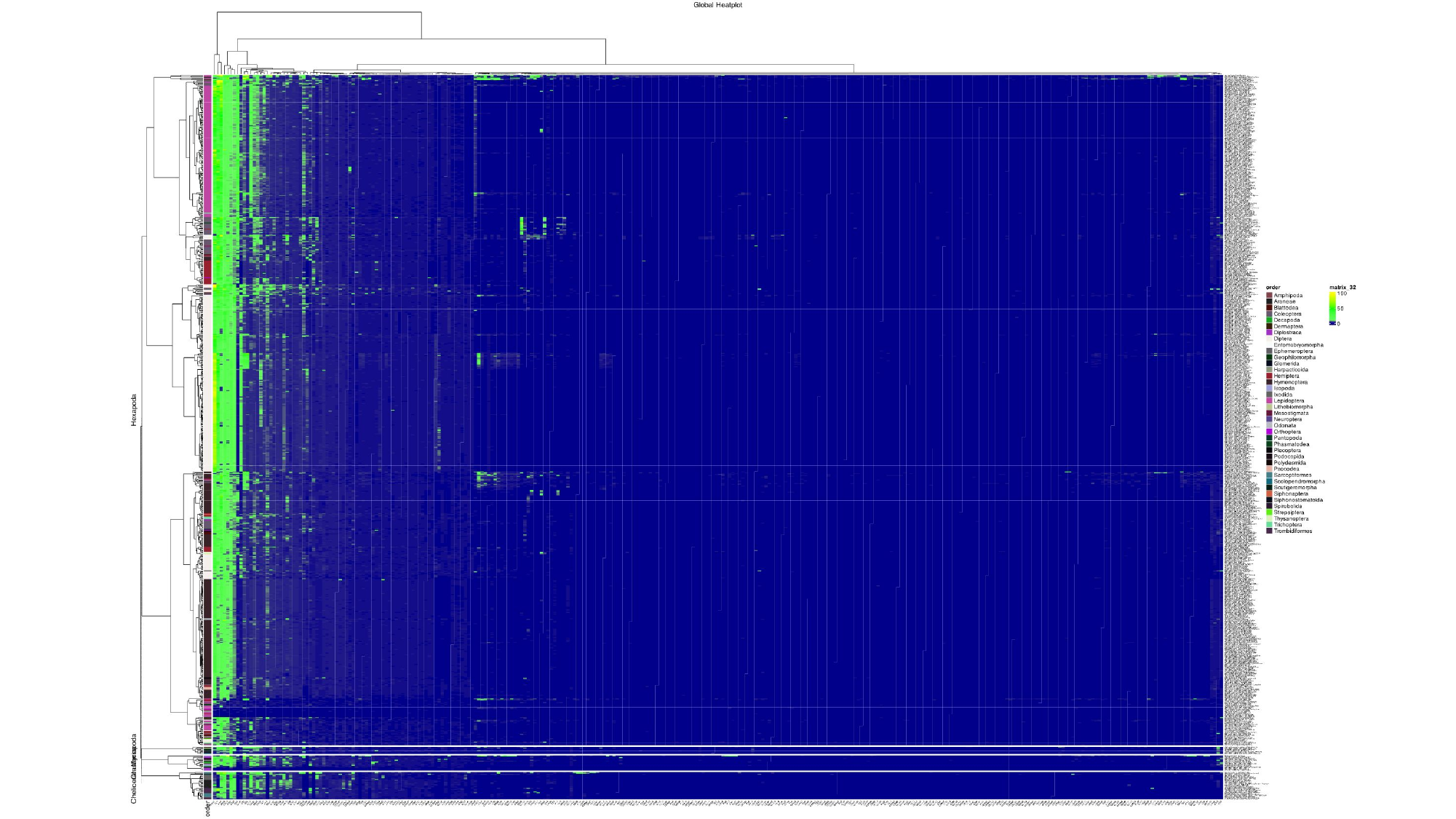

Supplement: Supplementary file 4 — Supplementary material [file mmc4.pptx]
